# Establishment of high reciprocal connectivity between clonal cortical neurons is regulated by the Dnmt3b DNA methyltransferase and clustered protocadherins

**DOI:** 10.1186/s12915-016-0326-6

**Published:** 2016-12-02

**Authors:** Etsuko Tarusawa, Makoto Sanbo, Atsushi Okayama, Toshio Miyashita, Takashi Kitsukawa, Teruyoshi Hirayama, Takahiro Hirabayashi, Sonoko Hasegawa, Ryosuke Kaneko, Shunsuke Toyoda, Toshihiro Kobayashi, Megumi Kato-Itoh, Hiromitsu Nakauchi, Masumi Hirabayashi, Takeshi Yagi, Yumiko Yoshimura

**Affiliations:** 1 Section of Visual Information Processing, National Institute for Physiological Sciences, National Institutes of Natural Sciences, Okazaki, Aichi 444-8585 Japan; 2 AMED-CREST, AMED, 1-3 Yamadaoka, Suita, 565-0871 Osaka Japan; 3 National Institute for Physiological Sciences, Section of Mammalian Transgenesis, Center for Genetic Analysis of Behavior, Okazaki, Aichi 444-8787 Japan; 4 KOKORO-Biology Group, Laboratories for Integrated Biology, Graduate School of Frontier Biosciences, Osaka University, 1-3 Yamadaoka, Suita, Osaka 565-0871 Japan; 5 Bioresource Center, Gunma University Graduate School of Medicine, Maebashi, 371-8511 Japan; 6 Division of Stem Cell Therapy, Center for Stem Cell Biology and Regenerative Medicine, Institute of Medical Science, University of Tokyo, Tokyo, 108-8639 Japan; 7 Department of Genetics, Institute for Stem Cell Biology and Regenerative Medicine, Stanford University, 291 Campus Drive, Li Ka Shing Building, Stanford, CA 94305-5101 USA; 8 Department of Physiological Sciences, The Graduate University for Advanced Studies (SOKENDAI), Okazaki, Aichi 444-8585 Japan

**Keywords:** Barrel cortex, Spiny stellate cell, Clonal neuron, iPS cells, Connection specificity, Reciprocity, Dnmt3b, Clustered protocadherin

## Abstract

**Background:**

The specificity of synaptic connections is fundamental for proper neural circuit function. Specific neuronal connections that underlie information processing in the sensory cortex are initially established without sensory experiences to a considerable extent, and then the connections are individually refined through sensory experiences. Excitatory neurons arising from the same single progenitor cell are preferentially connected in the postnatal cortex, suggesting that cell lineage contributes to the initial wiring of neurons. However, the postnatal developmental process of lineage-dependent connection specificity is not known, nor how clonal neurons, which are derived from the same neural stem cell, are stamped with the identity of their common neural stem cell and guided to form synaptic connections.

**Results:**

We show that cortical excitatory neurons that arise from the same neural stem cell and reside within the same layer preferentially establish reciprocal synaptic connections in the mouse barrel cortex. We observed a transient increase in synaptic connections between clonal but not nonclonal neuron pairs during postnatal development, followed by selective stabilization of the reciprocal connections between clonal neuron pairs. Furthermore, we demonstrate that selective stabilization of the reciprocal connections between clonal neuron pairs is impaired by the deficiency of DNA methyltransferase 3b (Dnmt3b), which determines DNA-methylation patterns of genes in stem cells during early corticogenesis. Dnmt3b regulates the postnatal expression of clustered protocadherin (cPcdh) isoforms, a family of adhesion molecules. We found that cPcdh deficiency in clonal neuron pairs impairs the whole process of the formation and stabilization of connections to establish lineage-specific connection reciprocity.

**Conclusions:**

Our results demonstrate that local, reciprocal neural connections are selectively formed and retained between clonal neurons in layer 4 of the barrel cortex during postnatal development, and that Dnmt3b and cPcdhs are required for the establishment of lineage-specific reciprocal connections. These findings indicate that lineage-specific connection reciprocity is predetermined by Dnmt3b during embryonic development, and that the cPcdhs contribute to postnatal cortical neuron identification to guide lineage-dependent synaptic connections in the neocortex.

**Electronic supplementary material:**

The online version of this article (doi:10.1186/s12915-016-0326-6) contains supplementary material, which is available to authorized users.

## Background

The specificity of synaptic connections in the sensory cortex is fundamental for the proper processing of sensory information. Specific neural connections form according to neuron properties, such as the morphological type, laminar location, projection area, and similarity of sensory responsiveness [1–8]. These specific connections can be achieved through molecular target-recognition mechanisms as well as activity-dependent mechanisms [9–12]. Neurons arising from the same single radial glial cell, a principal progenitor cell capable of generating neurons and glia in the cortex, are preferentially connected in the postnatal cortex [13, 14], suggesting that cell lineage contributes to connection specificity. However, little is known about the developmental process of lineage-dependent connection specificity, or how clonal neurons, which are derived from the same neural stem cell, are stamped with the identity of their common neural stem cell and guided to form mutual synaptic connections.

In epigenetic transcriptional regulation, which is important for embryonic development, DNA-methylation patterns are determined by de novo methylation by the DNA methyltransferases Dnmt3a and Dnmt3b in the embryo [15–17]. In mice, Dnmt3b is transiently expressed during stem-cell differentiation [17, 18] and DNA methylation is inherited by cell progeny [19]. Thus, DNA methylation by Dnmt3b might lead to neural stem cell labeling, which allows the descendants of individual stem cells to recognize each other. We recently demonstrated that Dnmt3b predetermines the stochastic expression of clustered protocadherin (cPcdh) isoforms in various combinations [20]. In mice, 58 *Pcdh* genes, which encode the cell-adhesion membrane protein cPcdhs, are organized into three gene clusters, *Pcdh*-*α*, *Pcdh*-*β*, and *Pcdh*-*γ* [21, 22]. Each neuron expresses its own set of isoforms, about 15 of the 58 cPcdh-family isoforms [23–26]. It seems that cPcdh isoforms, which exhibit remarkable extracellular diversity, bind homophilically in an isoform-specific manner [27–29], suggesting that they are involved in the discrimination between self and other neurons [20, 30, 31]. Thus, cPcdh expression patterns predetermined by Dnmt3b-dependent methylation in clonal neurons might reflect the progenitor identity and contribute to the recognition of pre- and postsynaptic partners to guide lineage-dependent synaptic connections.

In this study, we investigated the properties of lineage-dependent neural connections and the process and mechanism of their establishment. To this end, we targeted local neural connections in the whisker-related barrel in the mouse somatosensory cortex. Layer 4 excitatory neurons within a barrel share sensory inputs from a single whisker, and they are commonly involved in information processing of the inputs. These neurons are synaptically connected with each other at a high frequency [4]. We here show that reciprocal neural connections are formed and selectively retained between clonal neurons, and that this connection specificity is lost in the absence of Dnmt3b or cPcdhs. Our results suggest that specific connections between clonal neurons are predetermined by Dnmt3b-dependent gene regulation prior to neural differentiation, and that cPcdhs contribute to postnatal cortical neuron identification to guide lineage-dependent synaptic connections.

## Results

### Normal maturation of induced pluripotent stem cell-derived cortical neurons in chimeric mice

**Fig. 1:**
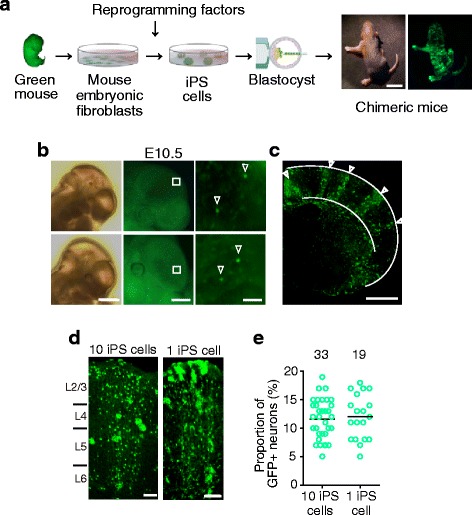
Visualization of clonal neurons using chimeric mice. **a** Production of chimeric mice from wild-type blastocysts and green fluorescent protein (GFP)-expressing induced pluripotent stem (iPS) cells. Scale bar: 10 mm. **b** Two examples of the cerebral vesicle in E10.5 chimeric mice observed by bright field (*left*) and fluorescence microscopy (*middle and right*). *Right panels* are magnified views of the boxed area in the middle panels. Arrowheads: GFP-labeled, iPS cell-derived stem cells. Scale bar: 1 mm (*left*), 500 μm (*middle*), 50 μm (*right*). **c** Distribution of GFP-positive cells in the neocortex of a postnatal day 10 (P10) wild-type chimeric mouse. Scale bar: 1 mm. **d** iPS-cell-generated GFP-positive cells in the barrel cortex of a P10 wild-type chimeric mouse generated by implanting 10 iPS cells (*left*) and one iPS cell (*right*). The neurons (cells with thin processes) and glial cells (cells with bushy processes) derived from iPS cells were visualized by GFP. Scale bar: 100 μm. **e** Proportion of GFP-positive neurons in the analyzed area in each column after the implantation of 10 iPS cells and one iPS cell. The number of tested columns is shown above the plots. A bar indicates mean value. No significant difference between these two groups was observed; *P* = 0.73 (*t* test)

To visualize clonal neurons derived from a single neural stem cell, we generated chimeric mice using induced pluripotent stem (iPS) cells marked with green fluorescent protein (GFP). We established several iPS cell lines from green mice (C57BL/6 background), in which all the cells express GFP [32], and then generated chimeric mice by injecting 10 iPS cells into the blastocysts of wild-type mice at embryonic day 3.5 (E3.5, Fig. 1a). Figure 1a shows a representative neonatal chimeric mouse with low GFP expression across the body surface. In the chimeric embryos showing relatively low expression of GFP across the body surface, the GFP-positive cells were very sparse in the cerebral vesicles at E10.5, early in corticogenesis (Fig. 1b), indicating that the GFP-positive cells appearing in the postnatal cortex would be derived from the small number of GFP-positive stem cells observed at E10.5 [33].

Around postnatal day 10 (P10), GFP-positive cells in the neocortex of low-GFP-expressing mice consisted of neurons and glial cells, and were distributed vertically through all layers in a columnar fashion across wide neocortical areas (Fig. 1c). In layer 4 of the barrel cortex, these GFP-positive neurons represented about 10% of the neurons within a labeled columnar area (Fig. 1d, e). We also established chimeric mice by injecting one iPS cell. These pups often showed undetectable GFP expression on the body surface. However, in the low-GFP-expressing mice, the proportion of GFP-positive cells in layer 4 was also about 10% (Fig. 1d, e). These observations are consistent with a previous study in chimeric mice generated using embryonic stem (ES) cells showing that a single neural stem cell in a cerebral vesicle around E10 produces clonal neurons accounting for about 10% of the neurons in the local cortical region [33]. Thus, in the low-GFP-expressing mice, the locally associated GFP-positive cortical neurons were likely generated from a neural stem cell that differentiated early in corticogenesis. We therefore used low-GFP-expressing chimeric mice in the following experiments.

**Fig. 2:**
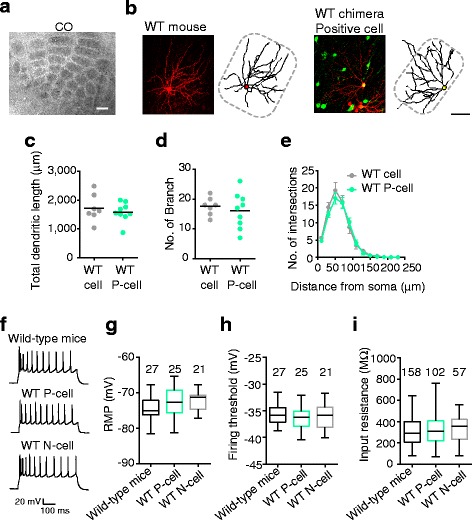
Morphological and electrophysiological properties of recorded layer 4 neurons. **a** Barrel structure visualized by cytochrome oxidase reaction (CO) in a chimeric mouse produced using green fluorescent protein (GFP)-positive wild-type induced pluripotent stem cells. Scale bar: 200 μm. **b** Spiny stellate cells (biocytin-stained, *red*), and traces of the soma and dendrites from a wild-type (WT) nonchimeric (*left*) and a GFP-positive wild-type chimeric mouse (*right*). Green: GFP-positive cells without biocytin injection. Dashed line: barrel. Scale bar: 50 μm. **c**–**e** Total dendritic length (c), number of dendritic branches (d), and Sholl analysis (mean ± SEM) (e) of spiny stellate cells in P18–20 wild-type nonchimeric mice (*gray*, n = 7 cells sampled from 3 mice) and chimeric mice (GFP-positive, *green*, n = 9 cells from 2 mice). A bar indicates the mean (c, d). **f** Representative traces of action potentials evoked by depolarizing current injection (300 pA) in current clamp mode in neurons from wild-type nonchimeric mice (WT, *upper*) and in GFP-positive (WT P-cell, *middle*) and GFP-negative neurons (WT N-cell, *lower*) from chimeric mice. **g**–**i** Box-and-whisker plots showing the value (median, 25th to 75th percentiles, minimum to maximum) of the resting membrane potential (RMP, g), the threshold to induce action potentials (firing threshold (h), and the input resistance (***i***) in each group at P18–20. The number of cells is indicated above each plot

Next, using barrel cortical slices with GFP-positive neurons distributed across layers in a column-like manner, we conducted whole-cell recordings from presumed excitatory layer 4 neurons. The barrel structures in layer 4 were normal in these mice (n = 3, Fig. 2a). We first examined whether the morphology of spiny stellate neurons, the major excitatory neurons in the layer 4 barrel, developed normally in these chimeric mice. We compared the morphology of GFP-positive spiny stellate neurons stained with biocytin after whole-cell recording in P18–20 chimeric mice with that of similarly treated spiny stellate neurons in age-matched wild-type nonchimeric mice (Fig. 2b and Additional file 1: Figure S1), and found no differences in total dendritic length (*t* test, *P* = 0.51; Fig. 2c), number of branches (*P* = 0.56; Fig. 2d), or expansion of dendritic territories (via a Sholl analysis; *P* > 0.21; Fig. 2e). Like the neurons in wild-type nonchimeric mice, the GFP-positive and -negative spiny neurons in the chimeric mice showed adaptive firing in response to depolarizing voltage steps (Fig. 2f), consistent with the previously reported firing patterns of layer 4 excitatory neurons [34]. No significant differences were found in the resting membrane potential (one-way ANOVA, *P* = 0.09; Fig. 2 g), threshold of action potential generation (*P* = 0.55; Fig. 2 h), or input resistance (*P* = 0.34; Fig. 2i) between these neuron groups. These results indicate that both the GFP-positive and -negative layer 4 excitatory neurons in the chimeric mice mature normally.

### Cell-lineage-dependent, high reciprocal connections between layer 4 excitatory neurons

**Fig. 3:**
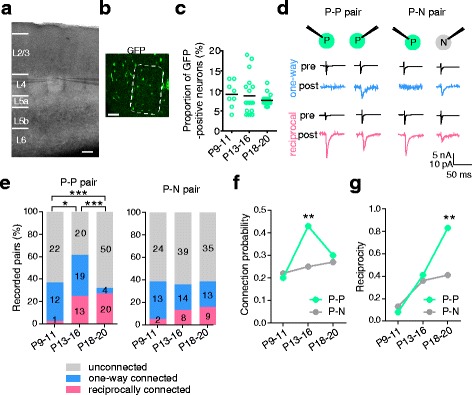
Synaptic connections between clonal and nonclonal neuron pairs in the barrel cortex. **a** Differential interference contrast image of a brain slice with two recording electrodes in a layer 4 barrel. Scale bar: 100 μm. **b** Green fluorescent protein (GFP)-positive cells in layer 4. Dashed line indicates a barrel. Scale bar: 50 μm. **c** Proportion of GFP-positive neurons in the analyzed area at each age. The number of tested columns is 8, 18, and 18 for P9–11, P13–16, and P18–20, respectively. *P* = 0.51 (Kruskal–Wallis test). **d** Representative average traces (n = 20) of action potentials evoked by brief depolarizing voltage pulses in one cell (pre) and resultant excitatory postsynaptic currents in the other (post) with one-way (*upper*) or reciprocal (*lower*) connections between GFP-positive excitatory neurons in layer 4 (P-P pair, *left*) and GFP-positive and -negative neurons (P-N pair, *right*) in P18–20 wild-type chimeric mice. **e** Percentage of P-P (*left*) and P-N (*right*) pairs with one-way (*blue*), reciprocal (*magenta*), or no (*gray*) connections; the number of recorded pairs is shown on each bar. Number of mice for P-P pairs: P9–11(n = 7), P13–16 (n = 10), P18–20 (n = 12); P-N pairs: P9–11(n = 6), P13–16 (n = 11), P18–20 (n = 9). *P < 0.05, ***P < 0.001: connection probabilities in different age groups (χ^2^ test with a 3 × 2 matrix after Bonferroni correction). **f**, **g** Developmental changes in the connection probability (f) and the proportion of reciprocal connections in connected pairs (reciprocity, g). **P < 0.01: P-P versus P-N pairs at the same age (Fisher’s exact test)

We examined the synaptic connections between GFP-positive neuron pairs (P-P pairs), which were presumed to be clonal neuron pairs, and between nonclonal GFP-positive and -negative neuron pairs (P-N pairs) using dual whole-cell recordings from layer 4 at P9–11, when synaptogenesis between layer 4 excitatory neurons starts in the barrel cortex [35], and at P13–16 and P18–20. We selected neuron pairs with an intercellular distance <50 μm within a barrel (Fig. 3a, b). Excitatory neurons in the layer 4 barrel consist of spiny stellate neurons and star pyramidal neurons. The recorded neurons visualized successfully by biocytin were mostly spiny stellate neurons (95%, n = 75 neurons) with a few star pyramidal neurons (5%, n = 4 neurons), indicating that the recorded neurons were excitatory neurons. In each age group, the GFP-positive neurons represented about 10% of all of the neurons in layer 4 within 200 μm of the recorded neurons (Fig. 3c).

We examined the connections between simultaneously recorded neurons in both directions. Figure 3d shows examples of one-way and reciprocal connections in P-P and P-N pairs. Among the P-P pairs, the proportion of three types of pairs (unconnected pairs and connected pairs with one-way connections, and reciprocal connections) changed significantly during the developmental stages examined, as tested using a χ^2^ test with a 3 × 2 matrix after Bonferroni correction (*P* < 0.05 for P9–11 versus P13–16; *P* < 0.001 for P9–11 versus P18–20 and for P13–16 versus P18–20; Fig. 3e). At P9–11, about a third of the pairs showed one-way connections, while reciprocal connections were rare. The proportion of reciprocally connected pairs increased from P9–11 to P13–16, while the proportion of one-way-connected pairs remained almost unchanged. If the probability of connection in one direction is independent of the presence or absence of connection in the other direction, the probability of reciprocal connections is expected to be the square of the connection probability. The proportion of reciprocal connections at P13–16 (25%) was higher than the expected proportion (18%), suggesting that reciprocal connections were preferentially established from P9–11 to P13–16. Thereafter, the proportion of one-way-connected pairs decreased, whereas that of reciprocally connected pairs remained almost the same. Such clear, age-dependent changes were not observed among the P-N pairs (Fig. 3e). Although the proportion of pairs with one-way, reciprocal, and no connections among P-N pairs was almost the same as that among P-P pairs at P9–11 (χ^2^ test with a 3 × 2 matrix, *P* = 0.89), it remained unchanged during the next periods (*P* = 0.29 for P9–11 versus P13-16; *P* = 0.92 for P13–16 versus P18–20).

To further characterize the developmental changes in these connections, we calculated the connection probability, defined as the number of detected connections divided by the total number of tested connections (Fig. 3f). The connection probability was not significantly different between P-P and P-N pairs at P9–11 (Fisher’s exact test, *P* = 0.84). At P13–16, the probability in P-P pairs increased more than 2-fold (*P* = 0.002) but that in P-N pairs remained almost the same; thus, P-P pairs connected more frequently than P-N pairs (*P* = 0.004). Thereafter, the connection probability became indistinguishable between P-P and P-N pairs at P18–20 (*P* = 0.68) as observed initially, owing to the reduction of one-way connections in P-P pairs (Fig. 3f). Thus, the connection probability appeared to increase transiently between clonal neuron pairs but remained almost unchanged between nonclonal neuron pairs during this period.

We also analyzed the reciprocity, which is the proportion of reciprocally connected pairs among connected pairs, and found clearly different developmental changes in the P-P versus P-N pairs. The reciprocity increased similarly in P-P and P-N pairs from P9–11 to P13–16. Thereafter, the reciprocity continued to increase in P-P pairs, whereas it remained almost unchanged in P-N pairs (Fig. 3 g). Consequently, at P18–20, reciprocal connections accounted for the majority of the connections between P-P pairs (83%) but for only 41% of those between P-N pairs (Fisher’s exact test, *P* = 0.005; Fig. 3 g). Thus, most of the connections between clonal neurons became reciprocal by 3 weeks after birth. In P-P pairs, high connection probability at P13–16 and high reciprocity at P18–20 were commonly observed in most of the animals (Additional file 2: Figure S2).

We examined whether there was any change in the morphological or electrophysiological properties of the spiny stellate cells between P13–16 and P18–20 when the dynamic, developmental change of the connectivity was observed in P-P pairs. No significant changes were found in the dendritic morphology of layer 4 spiny stellate neurons from P13–16 to P18–20 except that the number of branches very close to the soma increased (Additional file 3: Figure S3A–D). The small increase of branches near the soma might contribute to the transient increase of synapses in P-P pairs. In either the GFP-positive or GFP-negative neurons in chimeric mice, the resting membrane potential and threshold of action potential generation did not change from P13–16 to P18–20 (Additional file 3: Figure S3E, F). The input resistance decreased during this developmental period, consistent with a previous study (Additional file 3: Figure S3G) [36].

To examine whether the different developmental changes in P-P and P-N pairs resulted from an impaired connection development in chimeric mice, we compared the synaptic connectivity in chimeric and nonchimeric mice. The discrimination of P-P pairs from P-N pairs was not possible in nonchimeric mice, because the clonal neurons were not GFP-labeled. However, most of the cell pairs sampled from nonchimeric mice were probably P-N pairs, because clonal neurons originating from a single neural stem cell accounted for only about 10% of the neurons. The proportion of pairs with one-way, reciprocal, and no connections in nonchimeric mice (Additional file 4: Figure S4) was similar to that observed among the P-N pairs in chimeric mice at both P13–16 (χ^2^ test with a 3 × 2 matrix, *P* = 0.76) and P18–20 (*P* = 0.17) (Fig. 3e), indicating that the neural connections developed normally in the chimeric mice.

The chimeric mice used were mostly generated by injecting iPS cells derived from C57BL/6 mice into BDF1 blastocysts, although we used chimeric mice originating from C57BL/6 blastocysts in some of the experiments conducted at P18–20. Thus, the GFP-positive and GFP-negative neurons were derived from different mouse strains in most experiments. Although this difference might have caused the connectivity to differ between P-P pairs and P-N pairs, the connectivity at P18–20 was similar in chimeric mice generated using C57BL/6 blastocysts versus BDF1 blastocysts (Additional file 5: Figure S5A, B). This result suggests that the differences in connectivity arose from differences in cell lineage rather than in the mouse strains used for the iPS cells and blastocysts.

The connectivity difference between P-P and P-N pairs might also be ascribed to the fact that iPS cells are occasionally missing some genes [37]. However, we found similar high proportions of reciprocal connections in the P-P pairs at P18–20 for three different iPS cell lines (Additional file 5: Figure S5C). These results strongly support the view that reciprocal connections are frequently formed between layer 4 excitatory neurons originating from the same stem cell during normal development.

### Dnmt3b is required for cell-lineage-dependent high reciprocal connections

**Fig. 4:**
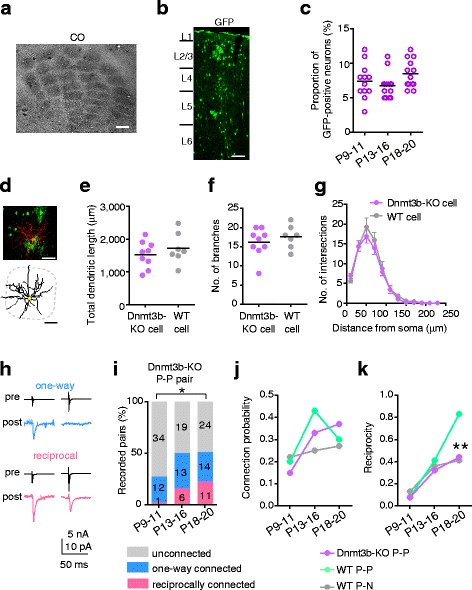
Effect of Dnmt3b deficiency on the morphology and synaptic connectivity of clonal neurons. **a** Barrel structure visualized by cytochrome oxidase reaction (CO) in a chimeric mouse produced using Dnmt3b-knockout (KO) induced pluripotent stem cells. Scale bar: 200 μm. **b, c** Distribution (b) and proportion (c) of Dnmt3b-KO cells in the analyzed area in each column. The number of columns is 12, 14, and 13 for P9–11, P13–16, and P18–20, respectively. *P* = 0.09 (Kruskal–Wallis test). Scale bar: 100 μm. **d** Dnmt3b-KO spiny stellate cell (*red, upper*) and traces of its soma and dendrites (*lower*). Scale bar: 50 μm. **e**–**g** Total dendritic length (e), number of dendritic branches (f), and Sholl analyses (mean ± SEM, g) of Dnmt3b-KO (*purple*, n = 9 cells sampled from 4 mice) and wild-type (WT; *gray*, the same as shown in Fig. 2) spiny stellate cells at P18–20. A bar indicates the mean (e, f). No significant differences in morphological parameters between these two groups were observed; *P* = 0.41 (e), *P* = 0.41 (f), and *P* > 0.14 (g) (*t* test). **h**–**k** Dual whole-cell recordings from Dnmt3b-KO GFP-positive excitatory neurons (*P-P*) pairs. **h** Representative average (n = 20) traces of presynaptic spikes (pre) and resultant excitatory postsynaptic currents (post) between Dnmt3b-KO P-P pairs with a one-way (*upper*) or reciprocal (*lower*) connection. **i** Percentage of Dnmt3b-KO P-P pairs with one-way (*blue*), reciprocal (*magenta*), or no (*gray*) connections. Numbers of recorded pairs are indicated on the bars. Number of mice: P9–11(n = 9), P13–16 (n = 11), P18–20 (n = 12). *P < 0.05: connection probabilities in different age groups (χ^2^ test with a 3 × 2 matrix after Bonferroni correction). **j**, **k** Connection probability (j) and reciprocity (k) of Dnmt3b-KO P-P (*purple*) and wild-type P-P (*green*) or GFP-positive and -negative neuron (P-N) (*gray*) cell pairs. **P < 0.01 compared with wild-type clonal pairs (Fisher’s exact test)

We next asked whether epigenetic regulation of gene expression is involved in lineage-dependent reciprocal connections. In mice, the DNA methyltransferase Dnmt3b is strongly expressed in neural stem cells at an early embryonic stage, but it disappears in the E13.5 cortex [15, 17, 18]. Thus, Dnmt3b-induced DNA methylation might be involved in the labeling of neural stem cells generated around E10. Because the DNA methylation pattern is inherited by the cell progeny [19], this labeling might contribute to the lineage-dependent connectivity in the postnatal cortex. To test this possibility, we produced chimeric mice using GFP-labeled iPS cells derived from Dnmt3b-knockout (KO) mice [20]. In these mice, the barrel structure was normal (n = 3, Fig. 4a), and the proportion of GFP-positive neurons in layer 4 of the barrel cortex was similar to that in the wild-type chimeric mice (Fig. 4b, c). Most of the recorded GFP-positive neurons were spiny stellate neurons (91%, n = 48; Fig. 4d), and a few were star pyramids (9%, n = 5). These spiny stellate neurons had dendritic morphologies similar to those observed in wild-type nonchimeric mice (Fig. 4e–g, Additional file 6: Figure S6A), and the electrophysiological membrane properties were similar between the GFP-positive neurons in wild-type and Dnmt3b-KO chimeric mice (Additional file 6: Figure S6B–E). These results indicated that the Dnmt3b-KO neurons developed normally.

At P9–11, the proportion of P-P pairs with one-way, reciprocal, and no connections in Dnmt3b-KO chimeric mice was similar to that in wild-type chimeric mice, and most of the connections were one-way (Fig. 4 h, i). Although the proportion of reciprocal, but not one-way, connections in Dnmt3b-KO P-P pairs increased to some extent from P9–11 to P13–16, the change was insignificant (χ^2^ test, *P* > 0.05), different from P-P pairs in wild-type chimeric mice. At P13–16, the proportion of pairs with one-way, reciprocal, and no connections observed among Dnmt3b-KO P-P pairs was not significantly different from that among wild-type P-N pairs (χ^2^ test with a 3 × 2 matrix, *P* = 0.37). The proportion remained unchanged from P13–16 to P18–20 (*P* = 0.70; Fig. 4i), in contrast to the notable decline in one-way connections observed in wild-type P-P pairs (Fig. 3e). There was no significant difference in the proportion between Dnmt3b-KO P-P pairs and wild-type P-N pairs at P18–20 (*P* = 0.43), indicating that Dnmt3b deficiency impaired the establishment of lineage-specific reciprocal connections at the later stage.

This difference in connectivity between Dnmt3b-KO and wild-type P-P pairs was clearly represented in the connection probability and reciprocity (Fig. 4j, k). The connection probability in Dnmt3b-KO P-P pairs was slightly smaller than that in wild-type P-P pairs at P9–11 (Fisher’s exact test, *P* = 0.41), and significantly increased from P9–11 to P13–16 (*P* = 0.006), with a rate similar to that found in wild-type P-P pairs (Fig. 4j). As a result, at P13–16, the connection probability was not significantly different from that in wild-type P-P pairs (*P* = 0.17) or in wild-type P-N pairs (*P* = 0.25), and was intermediate between the connection probabilities in wild-type P-P and P-N pairs. The connection probability continued to increase albeit insignificantly between P13–16 and P18–20 in Dnmt3b-KO P-P pairs (*P* = 0.63), unlike the steep decrease toward the initial value seen in wild-type P-P pairs. The reciprocity in Dnmt3b-KO P-P pairs at P9–11 (8%) and at P13–16 (32%) was almost the same as that observed in wild-type P-P pairs and P-N pairs at the same ages (Fig. 4 k). Notably, at P18–20, the reciprocity (44%) was significantly lower than that in wild-type P-P pairs (83%, *P* = 0.007) and was indistinguishable from that in wild-type P-N pairs (41%, *P* = 1.00; Fig. 4 k). These results were commonly obtained in most of the Dnmt3b-KO chimeric mice (Additional file 2: Figure S2). Thus, Dnmt3b deficiency impaired the transient increase in connections between clonal neuron pairs partially and the late removal of one-way connections totally, indicating that Dnmt3b is necessary for the development of cell-lineage-dependent reciprocal connections.

### Requirement of cPcdh for cell-lineage-dependent high reciprocal connections

The deletion of Dnmt3b causes hypomethylation of the promoter regions of *cPcdh-α, cPcdh-β*, and *cPcdh*-*γ* isoforms in the embryonic brain and in individual differentiated cerebellar Purkinje neurons [20]. The DNA methylation level of *cPcdh* genes in E9.5 embryos is the same as that in P21 cortex in wild-type mice and it is correlated with the expression level of each cPcdh isoform [20]. These findings suggest that if the Dnmt3b-dependent expression of cPcdh isoforms contributes to the establishment of lineage-dependent reciprocal connections, Dnmt3b deficiency might disrupt cPcdh expression, thereby abolishing the lineage-specific reciprocal connections between Dnmt3b-KO clonal cells. To test this possibility, we produced cPcdh-KO mice lacking all of the cPcdh isoforms (Additional file 7: Figure S7), and established chimeric mice using tdTomato-labeled iPS cells derived from the cPcdh-KO mice.

**Fig. 5:**
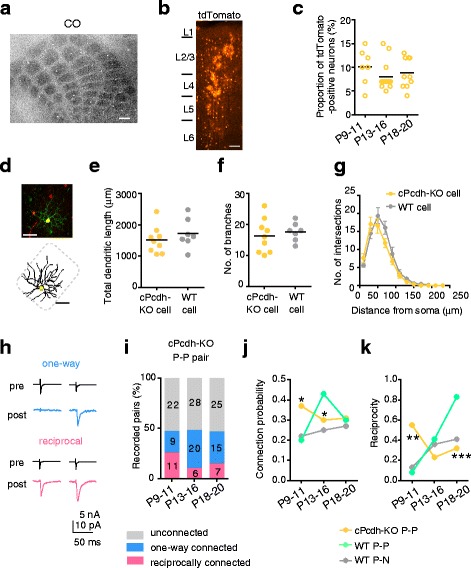
Effect of cPcdh deficiency on the morphology and synaptic connectivity of clonal neurons. **a** Barrel structure visualized by cytochrome oxidase reaction (CO) in a chimeric mouse prepared using cPcdh-knockout (KO) induced pluripotent cells. Scale bar: 200 μm. **b**, **c** Distribution (b) and proportion (c) of tdTomato-labeled cPcdh-KO cells in the analyzed area in each column. Number of columns is 7, 14, and 10 for P9–11, P13–16, and P18–20, respectively. *P* = 0.35 (Kruskal–Wallis test). Scale bar: 100 μm. **d** A cPcdh-deficient spiny stellate neuron (*yellow, upper*) and trace of its soma and dendrites (*lower*). Scale bar: 50 μm. **e**–**g** Total dendritic length (e), number of dendritic branches (f), and Sholl analyses (mean ± SEM, g) of cPcdh-KO (n = 9 cells sampled from 4 mice; *orange*) and wild-type (WT; *gray*, the same as shown in Fig. 2﻿) spiny stellate cells﻿ at P18–20. A bar indicates the mean (e, f). No significant differences in morphological parameters between cPcdh-deficient cells and wild-type cells were observed; *P =* 0.37 (e), *P =* 0.60 (f), and *P* > 0.07 (g) (*t* test). **h**–**k** Dual whole-cell recordings from cPcdh-KO GFP-positive excitatory neurons (P-P) pairs. **h** Representative average (n = 20) traces of presynaptic spikes (pre) and resultant excitatory postsynaptic currents (post) between cPcdh-KO P-P pairs with one-way (*upper*) or reciprocal (*lower*) connections. **i** Percentage of cPcdh-KO P-P pairs with each connection type. Number of recorded pairs is indicated on each bar. Number of mice: P9–11(n = 5), P13–16 (n = 11), P18–20 (n = 9). Developmental changes in connection probability (j) and reciprocity (k) in cPcdh-KO P-P (*orange*) and wild-type P-P (*green*) or or GFP-positive and -negative neuron (P-N; *gray*) cell pairs. *P < 0.05, **P < 0.01, ***P < 0.001 compared with wild-type clonal cell pairs (Fisher’s exact test)

The barrel structure developed normally in cPcdh-KO chimeric mice (n = 3, Fig. 5a) and the proportion of tdTomato-positive neurons in layer 4 of the barrel cortex was similar to that of GFP-positive neurons in the wild-type chimeric mice (Fig. 5b, c). Most of the tdTomato-positive neurons recorded from the layer 4 barrel were spiny stellate neurons (89%, n = 75); the rest were star pyramidal neurons (11%, n = 9). The dendritic morphology of the cPcdh-deficient spiny stellate neurons was similar to that observed in wild-type nonchimeric mice (Fig. 5d–g, Additional file 8: Figure S8A). The electrophysiological membrane properties of cPcdh-KO and wild-type GFP-positive neurons were similar (Additional file 8: Figure S8B–E). These results indicate that the cPcdh-KO neurons developed normally.

The development of synaptic connections between cPcdh-KO P-P pairs proceeded considerably differently from that between wild-type P-P pairs. At P9–11, the proportion of pairs with one-way, reciprocal, and no connections was significantly different between cPcdh-KO and wild-type P-P pairs (χ^2^ test with a 3 × 2 matrix, *P* = 0.02). The proportion among cPcdh-KO P-P pairs did not show any significant changes from P9–11 to P13–16 (*P* = 0.09) or from P13–16 to P18–20 (*P* = 0.79; Fig. 5 h, i). The proportion among cPcdh-KO P-P pairs was not significantly different compared with wild-type P-P (*P* = 0.14) or P-N pairs (*P* = 0.26), at P13–16. At P18–20, the proportion among cPcdh-KO P-P pairs was significantly different from that among wild-type P-P pairs (*P* = 0.0004) but not wild-type P-N pairs (*P* = 0.58). We confirmed that no significant differences were present in neural connectivity between cPcdh-KO chimeric mice generated using two different iPS cell lines (Additional file 8: Figure S8F). The connection probability for cPcdh-KO P-P pairs was highest at P9–11 and decreased slightly from P9–11 to P13–16 (Fisher’s exact test, *P* = 0.35), with no further change at P18–20 (*P* = 0.88; Fig. 5j). Compared with the wild-type P-P pairs at comparable ages, the connection probability in cPcdh-KO P-P pairs was about 2-fold higher (*P* = 0.03) at P9–11, significantly lower at P13–16 (*P* = 0.046), and similar at P18–20 (*P* = 0.89; Fig. 5j).

The reciprocity in cPcdh-KO P-P pairs showed a developmental change very different from that in wild-type P-P and P-N pairs (Fig. 5 k). The reciprocity was highest at P9–11 and decreased from P9–11 to P13–16 (Fisher’s exact test, *P* = 0.04) with no further change at P18–20 (*P* = 0.53), in contrast to the continuous increase throughout this period in wild-type P-P pairs. The reciprocity in cPcdh-KO P-P pairs was significantly higher than in both wild-type P-P (*P* = 0.009) and P-N pairs (*P* = 0.02) at P9–11, whereas at P18–20, it was significantly lower than that in wild-type P-P pairs (*P* = 0.0008) and almost the same as that in wild-type P-N pairs (*P* = 0.76). These developmental changes were commonly observed in most of the cPcdh-KO chimeric mice (Additional file 2: Figure S2). These results indicate that the cPcdh deficiency impaired the development of connections between P-P pairs more severely than the Dnmt3b deficiency, while the selective removal of one-way connections did not occur with either deficiency. Thus, it is likely that cPcdh is indispensable for the whole process by which lineage-dependent connection specificity is established, and that Dnmt3b is mainly required for the removal of one-way connections between clonal neurons.

### Regulation of the cPcdh expression pattern by Dnmt3b in barrel cortical neurons

We finally asked whether Dnmt3b indeed regulates the expression of cPcdh isoforms in layer 4 neurons of the barrel cortex. The expression pattern of cPcdh isoforms in clonal neurons was compared between wild-type and Dnmt3b-KO chimeric mice by in situ hybridization. We observed the mRNA signal for a single cPcdh isoform. In addition, we labeled all of the cells by Hoechst 33342 and clonal cells by immunostaining with an anti-GFP antibody. Among the cPcdh isoforms, γA3 and γA7 were selected for the analysis because of the high detection efficiency of their probes.

**Fig. 6:**
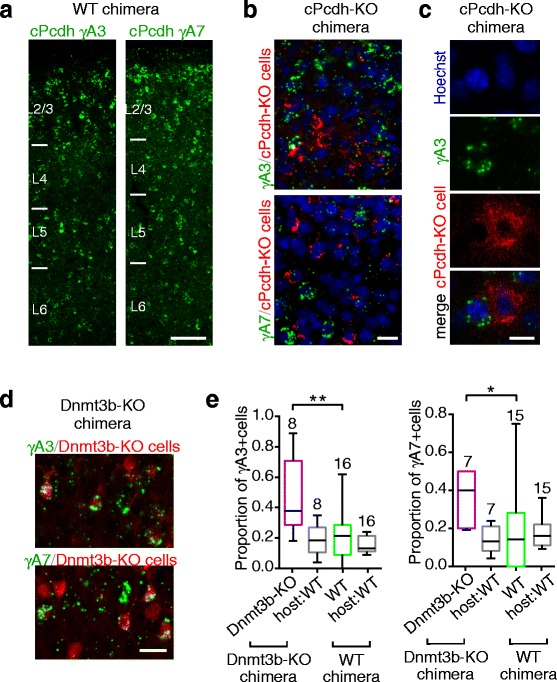
Effect of Dnmt3b deficiency on the expression of cPcdh isoforms. **a** Distribution of mRNA signals (*green*) for γA3 (*left*) and γA7 (*right*) in wild-type (WT) chimeric mice. Scale bar: 100 μm. **b**, **c** mRNA signals (*green*) for γA3 (*upper*) and γA7 (*lower*) in cPcdh-knockout (KO) chimeric mice (b) and γA3 expression at higher magnification (c). All cells and cPcdh-KO cells were visualized by Hoechst 33342 staining (*blue*) and a probe for the *td-Tomato* gene (*red*), respectively. Scale bar: 20 μm (b) and 10 μm (c). **d** Distribution of mRNA signals (*green*) for γA3 (*upper*) and γA7 (*lower*) in clonal Dnmt3b-KO cells visualized by immunostaining with an anti-green fluorescent protein (GFP) antibody (*red*) in Dnmt3b-KO chimeric mice. Scale bar: 20 μm. **e** Proportion of γA3-positive (*left graph*) and γA7-positive (*right graph*) neurons in induced pluripotent stem cell-derived Dnmt3b-KO cells (*magenta*) and wild-type cells (*green*) observed in slices prepared from Dnmt3b-KO chimeric mice and wild-type chimeric mice, respectively. The proportion of host-derived wild-type neurons that expressed γA3 or γA7 is shown in *gray*. The number of analyzed slices is shown above each box-and-whisker plot (median, 25th to 75th percentiles, minimum to maximum). *P < 0.05, **P < 0.01 (Mann–Whitney *U* test)

The cPcdh isoforms of γA3 and γA7 were both sparsely expressed in the barrel cortical cells in wild-type chimeric mice (Fig. 6a), consistent with our previous observation in wild-type non-chimeric mice [38]. Using cPcdh-KO chimeric mice, we confirmed the reliability of our method by comparing the mRNA signals for each cPcdh isoform between cPcdh-KO (tdTomato-positive) and wild-type (tdTomato-negative) cells (Fig. 6b, c). The mRNA signals for each cPcdh isoform were rarely detected in the tdTomato-positive cPcdh-KO cells, but were clearly detected in a portion of the adjacent tdTomato-negative wild-type cells.

To assess the proportion of cPcdh-expressing neurons, the neurons were morphologically discriminated from glial cells (see Methods). The proportion of *γ*A3-expressing neurons among Dnmt3b-KO clonal neurons (GFP-positive) in Dnmt3b-KO chimeric mice was about twice that among wild-type clonal neurons (GFP-positive) in wild-type chimeric mice (Mann–Whitney *U* test, *P* = 0.008), whereas no significant difference was found in the proportion of host neurons (GFP-negative) that expressed *γ*A3 between the two types of chimeric mice (*P* = 0.33, Fig. 6d, e). Similar results were obtained for the γA7 isoform (*P* = 0.03 for GFP-positive neurons, *P* = 0.50 for GFP-negative neurons; Fig. 6d, e). Thus, Dnmt3b deficiency caused an increase in cPcdh expression in barrel cortical neurons. These results suggest that Dnmt3b epigenetically regulates cPcdh expression, so that each group of clonal neurons expresses restricted subtypes of cPcdh isoforms.

## Discussion

Here we found that the synaptic connections between clonal neurons in layer 4 of the barrel cortex became mostly reciprocal when they were connected during postnatal development, and that the postnatal establishment of these connections required Dnmt3b and cPcdhs. Our analyses suggest that the Dnmt3b-mediated regulation of gene expression during embryonic development predetermines lineage-specific connectivity in the postnatal cortex and that the cPcdhs are involved in the total developmental process for establishing lineage-dependent connection specificity.

### Advantages of chimeric mice and the origin of GFP-positive neurons

To examine cell-lineage-dependent connections, we used chimeric mice generated using iPS cells marked with fluorescent proteins. This system also allowed us to delete a particular molecule from only the iPS cell-derived clonal neurons, and to analyze the effect of the deletion on lineage-dependent synaptic connections. Although Dnmt3b-KO mice and cPcdh-KO mice are embryonic lethal [15] and perinatal lethal (Hasegawa et al., personal communication), respectively, chimeric mice generated using iPS cells derived from these KO mice grew well, allowing us to analyze neural circuits composed of KO neurons. Because iPS cells are occasionally missing some genes [37], an unexpected deficiency of genes might affect neural connectivity between clonal neurons labeled using iPS cells. To avoid this possibility, we examined synaptic connections in chimeric mice generated using two to three different iPS cell lines and confirmed similar neural connectivity between them.

The proportion of cells derived from iPS cells in chimeric mice depends on the number of implanted and surviving iPS cells. Under our experimental conditions, the slices chosen for electrophysiological analyses included GFP-positive or tdTomato-positive neurons and glial cells that were distributed both locally and vertically though all layers in the barrel cortex. The distribution and proportion of fluorescently labeled cells within a column were indistinguishable between chimeric mice receiving one and 10 iPS cell implants, and almost the same as those of cortical neurons arising from a single stem cell differentiated early in corticogenesis [33, 39, 40]. Taken together, the fluorescently labeled neurons analyzed here appeared to originate from a single neural stem cell that differentiated at the early stage of corticogenesis.

### Developmental process for establishing lineage-specific connections

We found no difference in synaptic connectivity between clonal and nonclonal neuron pairs at the time point when synaptogenesis between cortical neurons normally starts [35]. In clonal, but not nonclonal, neuron pairs, we observed a transient increase in the proportion of connected pairs, and then a selective removal of one-way connections, resulting in high connection reciprocity, during the developmental period when dendritic spines are actively formed and eliminated [41, 42]. These results suggest that the high reciprocity in clonal pairs is attained by two sequential processes: an initial addition of new connections, which establishes reciprocal connections preferentially; and a subsequent selective retention of reciprocal connections.

### Molecular mechanisms for establishing lineage-dependent connections

A previous study demonstrated that cortical neurons arising from a single radial glial progenitor cell, called sister neurons, are more frequently connected with each other than nonsister neurons [13]. In the present study, we targeted neurons residing in the same layer and characterized postnatal developmental changes of neural connectivity that occurred specifically in clonal neuron pairs. The visualized clonal neurons presumably arose from a single neural stem cell that differentiated earlier than radial glial cells generating sister neurons [13, 33, 40]; this view is supported by the greater number of fluorescently labeled neurons in the present study than in the previous one. In both studies, neurons originating from the same progenitor cell were more specifically connected with each other than neurons originating from different progenitor cells. In the postnatal visual cortex, progeny neurons fluorescently labeled at early versus late stages of corticogenesis share a selective responsiveness to similar stimuli [39, 43]. Therefore, the mechanisms for establishing lineage-dependent connections may be at least partially common to groups of neurons originating from the stem cells differentiated at different embryonic stages. In the previous study, sister neurons formed a transient electrical coupling prior to establishing synaptic connections [14]. In this study, we found that cPcdh was required for lineage-specific reciprocal connections. To our knowledge, although the role of cPcdh in establishing gap junctions has not been determined, gap junctions might also help to establish lineage-dependent synaptic connections between clonal neurons in layer 4.

The present study showed for the first time that the epigenetic regulation of gene expression in a neural stem cell was required for the postnatal establishment of lineage-dependent connections. The DNA-methylation patterns determined by Dnmt3b can be inherited by progeny cells [19]. In mice, Dnmt3b is transiently expressed during stem-cell differentiation but disappears at a late stage of corticogenesis [15, 17, 18]. Thus, Dnmt3b-induced DNA methylation may be involved in labeling the lineage of clonal cells derived from a neural stem cell differentiated during early corticogenesis. The deficiency of Dnmt3b resulted in a complete failure to eliminate one-way connections established between clonal neurons, and it suppressed the initial increase in synaptic connections between clonal neurons to some extent. These findings suggest that the Dnmt3b-dependent epigenetic memory in individual stem cells may be necessary for establishing lineage-dependent reciprocal connectivity in the postnatal cortex, which occurs mainly through the selective removal of one-way connections.

It has been suggested that cPcdh is involved in the formation or elimination of synaptic connections. For example, deleting all of the *Pcdh*-γ isoforms inhibits synaptic formations in the spinal cord [44], while the expression of only one type of *Pcdh*-γ isoform in retinal starburst amacrine cells impairs the pruning of redundant synapses between these cells [31]. Even when the *Pcdh*s were totally deleted in the current study, synaptic connections were formed between clonal neurons at a proportion close to that observed in the presence of these genes. However, both the connection probability and connection reciprocity in these neurons were highest at the initial stage, and they decreased with development. These findings suggest that cPcdhs finely regulate the rate and timing of the synaptic formation and elimination between clonal neurons, although are not indispensable for the simple formation of connections. The earlier appearance of the peak in the connection probability and reciprocity in cPcdh-KO cell pairs might have resulted from the disorder of this regulation. Because cPcdhs interact with multiple other proteins and regulate intracellular signaling pathways, they can regulate synaptic formation or elimination through these pathways [44–46]. A deficiency in cPcdhs might cause the abnormal formation of connections between clonal neurons through the alteration in these signaling pathways.

The heteromer of cPcdh isoforms functions as a homophilic binding unit that induces a cell–cell interaction at the cellular membrane [28, 29]. If a particular combination of cPcdhs is selectively expressed in each group of clonal neurons in the barrel cortex as a result of Dnmt3b-induced DNA methylation during early corticogenesis, the homophilic interactions between cPcdh heteromers may play a crucial role in the selective removal of one-way connections between clonal neurons. In line with this hypothesis, Dnmt3b deficiency increased the proportion of clonal neurons expressing each cPcdh isoform in the barrel cortex, as observed previously in cerebellar Purkinje cells [20]. This condition may disturb the expression pattern of cPcdh isoforms in each group of clonal neurons. We observed that deficiencies of cPcdh and Dnmt3b both reduced the reciprocity in P-P pairs to the level in P-N pairs and hence caused clonal neurons to fail to establish lineage-specific connection reciprocity, suggesting that the expression pattern of cPcdh isoforms – presumably regulated by Dnmt3b – is important for the selective maintenance of reciprocal connections. On the other hand, we observed that the peak value of the connection probability in wild-type P-P pairs was slightly higher than that in Dnmt3b-deficient P-P pairs at the early stage, whereas it was far lower than that in cPcdh-deficient P-P pairs. Therefore, we cannot rule out the possibility that the regulation of cPcdh isoform expression by mechanisms other than Dnmt3b contributes to the establishment of reciprocal connections, especially the initial increase in synaptic connections between clonal neurons [47, 48]. In addition, other genes methylated by Dnmt3b can contribute to lineage-specific connectivity, because Dnmt3b is known to regulate the expression of many genes [49]. To verify our hypothesis, it will be necessary to comprehensively analyze the expression of multiple cPcdh isoforms in clonal and nonclonal neurons.

### Functional significance of lineage-dependent specific connections

In the visual cortex, the orientation selectivity of cortical neurons is already established by the time the eyes open [50, 51], and clonal neurons respond selectively to a similar orientation of visual stimulation [39, 43]. Therefore, it is likely that the cell-lineage-dependent establishment of neuronal connections is one of the mechanisms for the initial wiring of cell assembly underlying the selective responsiveness of cortical neurons before sensory experience.

Previous studies in layer 4 spiny stellate neurons of the barrel cortex demonstrated that the proportion of reciprocally connected pairs was consistent with that expected from the probability of unidirectional connection, assuming that the probability of connection in one direction is independent of the presence or absence of connection in the other direction [4, 34]. However, we found that the probability of reciprocal connections between clonal neurons was much higher than that expected from the probability of unidirectional connection. In the visual cortex, adjacent layer 2/3 pyramidal neurons with similar visual responsiveness are preferentially connected, and visual responses are more similar in reciprocally connected pairs than in pairs with a one-way connection [5]. In addition, these feature-specific connections mature without visual experience to some extent [52]. Taken together, in addition to activity-dependent mechanisms, cell-lineage-specific reciprocal connections may be involved in organizing microcircuits composed of neurons with similar sensory responsiveness.

Dnmt3b and cPcdh are candidate proteins involved in schizophrenia, bipolar disorder, and autism [53–55]. Thus, deficiencies in lineage-specific neural connections may play a role in these neuropsychiatric disorders, and further investigation into the underlying mechanisms of lineage-dependent connectivity may shed light on the pathology of these diseases.

## Conclusions

Our results demonstrate that local, reciprocal neural connections are selectively formed and retained between clonal neurons in layer 4 of the barrel cortex during postnatal development, and that Dnmt3b and cPcdhs are required for the establishment of lineage-specific reciprocal connections. These findings indicate that lineage-specific connection reciprocity is predetermined by Dnmt3b during embryonic development, and suggest that the Dnmt3b-dependent expression of cPcdh isoforms can at least partly contribute to postnatal cortical neuron identification to guide lineage-dependent synaptic connections in the neocortex.

## Methods

### Animals

All experiments were approved by the Animal Experiment Committees of the National Institute for Physiological Sciences, Osaka University and University of Tokyo. The mice were raised on a 12-h light/dark cycle with water and food ad libitum. Two to seven mice were housed in the same cage.

### Generation of iPS cells and chimeric mice

To generate wild-type iPS cells, we used green mice backcrossed to C57BL/6 mice. The green mice ubiquitously express enhanced GFP driven by a cytomegalovirus early enhancer element and chicken beta-actin (CAG) promoter (kindly provided by Dr M Okabe of Osaka University) [32]. To generate Dnmt3b-KO iPS cells, *Dnmt3b* ^*del*^ heterozygous mice were crossed with the green mice, and Dnmt3b homozygous green embryos were used. For cPcdh-KO iPS cells, *Pcdh-abg* ^*del*^ heterozygous mice were crossed with *TG* ^*taf7*^ transgenic mice, and *Pcdh-abg* ^*del*^ homozygous and *TG* ^*taf7*^ transgene-positive embryos were used (Additional file 7: Figure S7). Fibroblasts were isolated from male embryos at E13.5. The iPS cells were generated by introducing retroviral vectors encoding reprogramming factors as described previously [56]. Genotyping was carried out by PCR on DNA extracted from iPS cells, and loss of Dnmt3b and cPcdh genes was confirmed in the iPS cells generated from *Dnmt3b* ^*del*^ and *cPcdh* ^*del*^ homozygous mice, respectively (Additional file 9: Figure S9). The primer sequences used for genotyping are shown in Additional file 10: Table S1. To generate chimeric mice, 10 iPS cells were injected into a C57BL/6 or BDF1 blastocyst, which was then transferred into the uterus of a pseudopregnant ICR female mouse. In some of experiments, one iPS cell was injected. The C57BL/6, BDF1, and ICR laboratory mice were purchased from SLC Japan.

### Generation of *Pcdh-abg*^*del*^ mice

An *a1MV* targeting vector was used to introduce a *loxP* site upstream of the *Pcdh*-*a* cluster and a Myc-tagged-venus fluorescent protein gene into the *Pcdha1* exon. The *a1MV* targeting vector was prepared by bacterial artificial chromosome (BAC) modification [57]. Purified mouse BAC RP23-303I8 bearing the *Pcdh-a1* gene was transferred into *Escherichia coli* EL350 cells. To construct the *a1MV* targeting vector, homologous fragments of the targeting sites were inserted into the pBTloxP2 plasmid, which contains a floxed *neo* ^*r*^ cassette. These homology arms were generated by PCR amplification using the mouse BAC as a template (Additional file 7: Figure S7). The *Acc*I fragment of the 5′ homology region, which was amplified by the a1MVA-F and a1MVA-R primers, was subcloned into the *Cla*I restriction site of pBTloxP2. The Myc-tagged-venus-fused 3′ homology fragment consisted of the *Cla*I-digested amplicon obtained using the a1MVB-F and a1MVB-R primers; the *Cla*I, *Sbf*I-digested amplicon obtained using the a1MVC-F and a1MVC-R primers with venus cDNA as a template; and the *Sbf*I-digested amplicon obtained using the a1MVD-F and a1MVD-R primers. The Myc-tagged-venus-fused 3′ homology fragment was subcloned into the *Bam*HI and *Sac*I restriction sites of pBTloxP2. The floxed *neo* ^*r*^ gene with the Myc-tagged-venus gene and homology arms was excised by *Sal*I digestion, and gel-purified. The purified *neo* ^*r*^ cassette was electroporated into EL350 cells containing RP23-303I8, which had been induced for the Red recombination functions by prior growth at 42 °C for 15 min [57]. Transformants were selected on plates containing kanamycin. The modification of the BACs was verified by PCR. Finally, using homologous recombination, the BAC DNA fragment containing the Myc-tagged venus and *neo* ^*r*^ gene was inserted into pBRSDT, which contains the gene for diphtheria toxin A in the *Sal*I site of pBR322 [58, 59]. The homology fragments for this recombination were amplified by the a1MVE-F and a1MVE-R primers and the a1MVF-F and a1MVF-R primers, and were subcloned into pBRSDT at the *Hin*dIII and *Nhe*I restriction sites. We retrieved the 16-kb BAC DNA fragments that contained the Myc-tagged venus and floxed *neo* ^*r*^ genes, and inserted them into pBRSDT. The retrieved plasmid was used as a targeting vector.

The linearized targeting vector was electroporated into TT2 ES cells, which were screened for the mutant allele by Southern hybridization analysis (Additional file 7: Figure S7) with a probe that was isolated by PCR using the mouse BAC as a template. The recombinant ES cell clones were injected into ICR blastocysts, and the male chimeras were bred with C57BL/6 mice. The primer sequences used to construct the targeting vector and to isolate the probes for Southern hybridization analysis are shown in Additional file 10: Table S1. The generation of *gLacZ* mutant mice, in which the *lacZ* gene and *loxP* site are inserted downstream of the gCR3 exon, has previously been described [60].

The cluster deletion allele *Pcdh-abg* ^*del*^ was generated by Cre-induced meiotic recombination between *a1MV* and *gLacZ* double-mutant mice and synaptosomal complex protein 1 (Sycp1)-Cre transgenic mice [61] (Additional file 7: Figure S7). Male mice that carried both the *a1MV* and *gLacZ* mutant alleles and the Sycp1-Cre transgene were crossed with C57BL/6 females, and the genotypes of the pups were determined by PCR. The primer sequences used for genotyping are shown in Additional file 10: Table S1.

### Generation of *TG*^*taf7*^ transgenic mice

A 108-kb DNA fragment containing the TAF7 gene, which are located upstream of the *Pcdh-g* cluster, was isolated from the mouse BAC RP23-440 M2 by BAC modification methods and was used as a transgene to generate *TG* ^*taf7*^ transgenic mice. Injection of the transgene into blastocysts of C57BL/6 mice, and the implantation of blastocysts into the uterus of ICR mice, were performed according to standard protocols [62]. The transgene was detected by PCR with a primer set (5′-GCGCGCCAAAGCTTGCATGC-3′ and 5′-CTCTCCCTATAGTGAGTC-3′) that amplified the DNA fragment corresponding to the BAC vector pBACe3.6.

### Slice preparations

Mice of either sex were used for electrophysiological and morphological experiments. Chimeric mice of either sex at different postnatal ages (P9–11, P13–16, and P18–20) were deeply anesthetized with sodium pentobarbital (50 mg/kg, intraperitoneal injection) and perfused transcardially with ice-cold normal artificial cerebrospinal fluid (ACSF) containing 126 mM NaCl, 3 mM KCl, 1.3 mM MgSO_4_, 2.4 mM CaCl_2_, 1.2 mM NaH_2_PO_4_, 26 mM NaHCO_3_, and 10 mM glucose, saturated with 95% O_2_ and 5% CO_2_. The perfused ACSF also contained 1 mM kynurenic acid to prevent the death of layer 4 cells. Wild-type C57BL/6 mice were used for some experiments. The brains were removed, and parasagittal (35–40 degrees away from vertical) slices (300 μm) of barrel cortex were cut using a vibrating microslicer (VT1200S; Leica) and recovered in an interface chamber at 33 °C for 1 h, as described previously [12]. The slices were then transferred to a submerged chamber containing normal ACSF oxygenated with 95% O_2_ and 5% CO_2_ at room temperature.

### Electrophysiology

In experiments using different kinds of chimeric mice, the recording experiments were performed in a blinded manner and a randomized order. Barrel cortical slices were transferred into a submerged chamber containing normal ACSF without kynurenic acid. Fluorescent protein-expressing and -nonexpressing neurons were identified under fluorescent and infrared differential interference contrast optics with an X40, 0.8NA water immersion lens (BX-50WI, Olympus). We chose slices with a relatively small number of GFP-positive neurons for recordings. Neuron pairs located in a single barrel of layer 4 with a distance between their somata of <50 μm were targeted for dual whole-cell recordings. Spiny stellate cells with small round cell bodies were targeted for recording. We recorded neurons with the soma located at least 50 μm below the cut surface of the slice. Patch pipettes (5–7 MΩ) were filled with a solution containing 130 mM K-gluconate, 8 mM KCl, 1 mM MgCl_2_, 0.6 mM EGTA, 10 mM HEPES, 3 mM MgATP, 0.5 mM Na_2_GTP, 10 mM Na-phosphocreatine, and 0.2% biocytin (pH 7.3 adjusted with KOH). The resting membrane potentials and firing patterns evoked by depolarizing current injections were measured in current clamp mode. To analyze synaptic connections, the membrane potential of the recorded cells was held at the reversal potential of inhibitory postsynaptic currents (−70 mV). In all paired recordings, the connections between neuron pairs were assessed in both directions by applying brief (1–2 ms) depolarizing voltage pulses (at least 50 trials) to evoke action potentials in one of the cells and recording excitatory postsynaptic currents from the other cell. For analysis, we selected cells with a series resistance <30 MΩ. We did not use series resistance compensation. All recordings were conducted using a Multiclamp 700B amplifier, and data were analyzed using pClamp9 software (Molecular Devices).

### Post hoc morphological analysis

After whole-cell recording, the slices were fixed with 4% paraformaldehyde (PFA) in 0.1 M phosphate buffer (PB, pH 7.4) overnight. To visualize the recorded neurons and the laminar structure, slices were incubated with streptavidin conjugated to Alexa 568 or 488 (1:1000; S11226, RRID: AB_2315774, S11223, RRID: AB_2336881, Life Technologies) in 25 mM PBS containing 0.1% Triton X-100 overnight at room temperature, followed by DAPI staining for 30 min. Images of slices were obtained using an A1R confocal microscope (Nikon) with a × 40 objective. The dendrite morphology was manually traced using Neurolucida software (MBF Bioscience). Spiny stellate neurons were distinguished from star pyramidal cells by their spherical somata and the absence of a prominent apical dendrite [63]. For counting of dendritic branches and Sholl analysis, the whole dendrites of biocytin-filled neurons were used. The total dendritic length was measured and Sholl analysis was performed using Neurolucida software (MBF Bioscience).

To calculate the proportion of iPS cell-derived cells, images of recorded slices were obtained using the confocal microscope with a × 40 objective and the fluorescent-protein-expressing cells in the area around the recorded neurons (200 × 200 μm) were counted manually using ImageJ software. In some slices, neurons were visualized by the immunostaining for NeuN to estimate the total number of neurons in the analyzed area in layer 4. The slices were incubated with mouse anti-NeuN antibody (1:1000; MAB377, RRID: AB_2298772, Millipore) in 25 mM PBS containing 2.5% normal goat serum, 0.1% Triton X-100, and 0.25% λ-carrageenan at 4 °C overnight. After several washes in 25 mM PBS, the slices were incubated with goat anti-mouse IgG antibody conjugated to Alexa 568 (1:1000; A11004, RRID:AB_2534072, Life Technologies) in 25 mM PBS containing 0.1% Triton X-100 for 2 h at room temperature. The number of NeuN-stained neurons was counted in the local area (200 × 200 μm) in layer 4. The average number of layer 4 neurons in the area was 177 ± 4.3, 174 ± 1.3, and 150 ± 9.1 (mean ± SEM) in P9–11, P14–16, and P18–21 mice, respectively (six slices in each group). For calculation of the proportion of iPS cell-derived cells, the number of fluorescent-protein-expressing cells was divided by the average number of NeuN-stained cells in the area (200 × 200 μm) and multiplied by 100.

### Cytochrome oxidase staining

Chimeric mice were perfused transcardially with 4% PFA in 0.1 M PB for 10 min. After decapitation, the cortex was isolated from the other brain regions and flattened between glass slides in the same fixative for 2–3 h. Slices were prepared in the tangential plane at 50-μm thickness using a vibrating microslicer (DTK-100; Dosaka). After several washes with 25 mM PBS, the slices were incubated in a solution containing 0.05% DAB, 0.02% catalase, and 0.03% cytochrome c in 25 mM PBS for 2–4 h.

### In situ hybridization and immunofluorescence staining

Antisense RNA probes for *PcdhγA3*, *PcdhγA7* [38], and *tdTomato* were synthesized using a digoxigenin (DIG) or fluorescein-RNA labeling kit with T3 or T7 RNA polymerase (Roche). To generate specific probes for individual *Pcdhγ* genes, we selected probe sequences that have low homology to any *Pcdhγ* genes except for the target gene. The alignment scores obtained using the ClustalW program are shown in Additional file 11: Table S2. Wild-type, *Dnmt3b*-KO, and *cPcdh*-KO chimeric mouse pups (P10) of either sex were used for dual fluorescent in situ hybridization (ISH) or simultaneous ISH/immunofluorescent (ISH/IF) staining. ISH was carried out on slide-mounted histological sections, using standard procedures with minor modifications [64]. Briefly, 30-μm-thick slide-mounted sections were permeabilized, digested with Proteinase K, and acetylated before prehybridization. They were then incubated in hybridization buffer [50% deionized formamide, 2% Blocking Reagent (BR; Roche), 5× Saline Sodium Citrate (SSC), 0.1% N-lauroylsarcosine, and 0.1% sodium dodecyl sulfate] at 60 °C for 2–4 h. They were then transferred to new hybridization buffer containing DIG-labeled and/or fluorescein-labeled heat-denatured antisense RNA probes and incubated at 60 °C overnight. After hybridization, the sections were washed and then incubated in 1% BR. After blocking, sections for (1) ISH/IF and (2) dual fluorescent ISH were processed differently as described below. The combinations of mouse strains, staining methods, RNA probe label, and antibodies used are shown in Additional file 12: Table S3.

#### I*n situ* hybridization/immunofluorescence

Sections for ISH/IF staining were incubated with an anti-fluorescein antibody conjugated with horseradish peroxidase (1:4000, Jackson ImmunoResearch Laboratory) and a chicken anti-GFP antibody (1:500; ab13970, RRID: AB_300798, Abcam) in BR overnight. The fluorescein signals were enhanced with the TSA Plus DNP System (PerkinElmer LifeSciences, Boston, MA, USA). The sections were washed with Tris NaCl Tween20 (TNT) buffer and then incubated with an anti-DNP antibody conjugated with Alexa Fluor 488 (1:500; A-11097, RRID: AB_2314332, Molecular Probes) and anti-chicken IgG conjugated with Alexa Fluor 594 (1:500; A-11042, RRID: AB_2534099, Molecular Probes) overnight for fluorodetection. After washing, the sections were counterstained with Hoechst 33342 (Sigma).

#### Dual fluorescent i*n situ* hybridization

Sections for dual fluorescent ISH were incubated with the anti-fluorescein antibody conjugated with horseradish peroxidase in BR overnight. The fluorescein signals were then enhanced with the TSA Plus DNP System. After washing, the sections were incubated with the anti-DNP antibody conjugated with Alexa Fluor 488 for fluorodetection, and the anti-DIG antibody conjugated with alkaline phosphatase overnight. The sections were washed with TNT and then with TS8.0 buffer (0.1 M Tris-HCl; 0.1 M NaCl; 10 mM MgCl_2_ pH 8.0). They were then transferred to HNPP/Fast Red TR (Roche) solution until adequate fluorescent color development. After washing, the sections were counterstained with Hoechst 33342.

#### Quantitative analysis

For quantitative analysis, three-dimensional images of the ISH samples were obtained using an A1R confocal microscope with a × 40 objective (Nikon). The layer 4 region in each GFP-positive clonal column (see Fig. 1c) was captured. Neurons were discriminated from glial cells or vascular cells based on differences in the shape of the nuclei shown by Hoechst 33342 staining and in the cell shape shown by GFP staining. The γA3- or γA7-positive neurons were counted in both the GFP-positive and -negative neurons within a single barrel. A very low number of fluorescent particles for γA3 or γA7 mRNA was detected around a nucleus of cPcdh-KO cells [1.25 ± 1.25 particles (mean ± SD) for γA3 (n = 56 cells) and 0.92 ± 1.13 particles for γA7 (n = 36 cells)]. We considered the γA3 or γA7 to be expressed in a neuron when the number of mRNA signals around a nucleus exceeded two SD from the average observed in cPcdh-KO cells (at least four particles of fluorescent label for γA3 or γA7). All of the counting was performed using ImageJ software.

### Statistical analysis

GraphPad Prism was used for the statistical analyses. For all of the experiments except for the probability of connection, the normality of the distribution and equality of variance were tested. A parametric two-tailed *t* test (for two groups) and one-way ANOVA followed by Tukey’s test (for more than two groups) were applied when the data passed these tests. Otherwise, a non-parametric Mann–Whitney *U* test (for two groups) and Kruskal–Wallis test (for more than two groups) were used. To compare the probability of connection, Fisher’s exact test was applied for the comparison of the proportion of connected pairs or reciprocity, and the χ^2^ test with a 3 × 2 matrix was used to compare the proportion of unconnected, one-way connected, and reciprocally connected pairs. *P* values <0.05 were considered significant for the comparison of two groups. The significance threshold was determined with Bonferroni correction for the comparison of more than two groups.

## Additional files

Additional file 1:
**Figure S1.** Dendritic morphology of recorded layer 4 neurons. (**A, B**) Traces from spiny stellate cells visualized by biocytin staining in layer 4 of the barrel cortex at P18–20. Images show seven neurons from wild-type nonchimeric mice (A), and nine GFP-positive neurons (P cells) from wild-type chimeric mice (B). Scale bar: 100 μm. (PDF 673 kb)
Additional file 2:
**Figure S2.** Synaptic connectivity obtained from each animal in four groups of neuron pairs. (**A–C**) Connection probability in wild-type (WT) P-P and P-N pairs, and Dnmt3b-KO and cPcdh-KO neuron pairs at P9–11 (A), P13–16 (B), and P18–20 (C). (**D–F**) Reciprocity in WT P-P and P-N pairs, and Dnmt3b-KO and cPcdh-KO neuron pairs at P9–11 (D), P13–16 (E), and P18–20 (F). Each symbol indicates an average value obtained from each animal. (PDF 416 kb)
Additional file 3:
**Figure S3.** Comparison of morphological and electrophysiological properties of layer 4 neurons between P13–16 and P18–20 chimeric mice. (**A**) Traces of spiny stellate cells sampled from P13–16 wild-type chimeric mice (n = 9 cells, n = 8 barrels, n = 3 mice). Scale bar: 100 μm. (**B–D**) Comparison of the dendritic morphology between P13–16 (n = 9) and P18–20 (n = 16) chimeric mice. No significant differences in the total dendritic length (*P =* 0.26, *t* test, B) or the number of branches (*P =* 0.20, C) were observed, but there was a difference in the number of intersections near soma (**P =* 0.046, D). A bar indicates the mean (B, C). Data presented as mean ± SEM in D. (**E–G**) Comparison of the GFP-negative neurons (N-cell) or GFP-positive neurons (P-cell) between P13–16 and P18–20 chimeric mice in the resting potential (*P* = 0.30 for GFP-negative neurons, *P* = 0.36 for GFP-positive neurons, *t* test, E), firing threshold (*P* = 0.44 for GFP-negative neurons, *P* = 0.20 for GFP-positive neurons, F), and input resistance (****P* = 0.0007 for GFP-negative neurons, ***P* = 0.003 for GFP-positive neurons, G). Number of analyzed cells is shown above each box-and-whisker plot (median, 25th to 75th percentiles, minimum to maximum) (E–G). (PDF 636 kb)
Additional file 4:
**Figure S4.** Synaptic connections in wild-type nonchimeric mice. (**A**) Representative average (n = 20) traces of presynaptic spikes (pre) and resultant excitatory postsynaptic currents (post) between layer 4 neuron pairs with a one-way (upper) or reciprocal (lower) connection in wild-type nonchimeric mice (C57BL/6 mice) at P18–20. (**B**) Percentage of neuron pairs with synaptic connections. There was no significant difference in the connectivity between the neuron pairs in wild-type nonchimeric mice and the nonclonal neuron pairs in wild-type chimeric mice at the same age (*P* > 0.17, χ^2^ test). Numbers of recorded pairs are indicated on the bars. (PDF 575 kb)
Additional file 5:
**Figure S5.** Synaptic connectivity is not affected by the mouse strain used for the blastocysts or iPS cell lines. (**A**) Percentage of synaptic connections between clonal neuron pairs (P-P pairs) in P18–20 wild-type chimeric mice produced with blastocysts prepared from BDF1 or C57BL/6 mice; iPS cells were produced using C57BL/6 mice. There was no significant difference in the connectivity between the two strains (*P* = 0.55, χ^2^ test). (**B**) Similar to A, but between nonclonal neuron pairs (P-N pairs). There was no significant difference in the connectivity between the two strains (*P* = 0.68, χ^2^ test). (**C**) Percentage of synaptic connections between GFP-positive neuron pairs (P-P pairs) in P18–20 chimeric mice produced from different wild-type iPS cell lines (iPS-1, iPS-2, and iPS-3). No significant difference was observed between any of the three iPS cell lines (*P* = 0.59, χ^2^ test). Numbers of recorded pairs are indicated on the bars. (PDF 540 kb)
Additional file 6:
**Figure S6.** Morphological and electrophysiological properties of Dnmt3b-KO neurons. (**A**) Traces from iPS cell-derived Dnmt3b-KO spiny stellate cells in layer 4 of the barrel cortex in Dnmt3b-KO chimeric mice at P18–20. The neurons were stained by biocytin. Scale bar: 100 μm. (**B**) A representative trace of action potentials evoked by depolarizing current injection (300 pA) in current clamp mode in Dnmt3b-KO neurons. (**C–E**) Box-and-whisker plot showing the value (median, 25th to 75th percentiles, minimum to maximum) of the resting membrane potential (RMP, C), the threshold to induce action potentials (firing threshold, D), and the input resistance (E) in Dnmt3b KO neurons (purple) and wild-type GFP-positive neurons (green, the same as shown in Fig. 2) at P18–20. No significant differences in electrophysiological parameters between Dnmt3b-deficient cells and wild-type cells were observed; *P =* 0.72 (C), *P =* 0.71 (D), and *P =* 0.68 (E) (*t* test). The numbers of cells are indicated above the box-and-whisker plot. (PDF 728 kb)
Additional file 7:
**Figure S7.** Generation of cPcdh KO mice that have a *Pcdh-abg* ^*del/del*^ allele and a *TG* ^*taf7*^ BAC transgene. (**A**) Schematic diagram of the *a1MV* targeting constructs. Filled triangles: *loxP* sites; open triangles: *frt* sites; M: Myc-tagged venus fluorescent protein gene; Neo: neomycin-resistance gene; DT-A: diphtheria toxin A fragment gene; α1: *cPchd-a1* exon; α2: *cPchd-a2* exon; α3: *cPchd-a3* exon; B: *Bam*HI; Sp: *Spe*I. (**B, C**) Southern blotting of homologous recombinant ES cells digested by *Bam*HI, with Probe A (B), and digested by *Spe*I, with Probe B (C). (**D**) Genetic structures of *loxP* site insertions. The *loxP* sites were inserted 5′ of the *Pcdh-α* cluster and 3′ of the *Pcdh-γ* cluster in *a1MV* and *gLacZ* mutant mice, respectively. (**E**) Genetic structures of *Pcdhαβγ* ^*del*^ mice and *TG* ^*taf7*^ BAC transgenic mice. Arrows indicate the primer positions used for genotyping. αCR: cPcdh-α constant region; γCR: cPcdh-γ constant region. (**F, G**) Genotyping by PCR of cPcdh-KO mutants with the *Pcdh-αβγ* ^*del*^ allele and the *TG* ^*taf7*^ BAC transgene. (**H**) Expression of *Pcdh-α*, *Pcdh-β*, *Pcdh-γ*, and *taf7* genes in the E18.5 brain of wild-type and cPcdh-KO mice, by RT-PCR analysis. All of the genotyping and gene expression experiments yielded similar results. (PDF 869 kb)
Additional file 8:
**Figure S8.** Morphological and electrophysiological properties of cPcdh-KO neurons. (**A**) Traces from iPS-derived tdTomato-positive spiny stellate cells in layer 4 of the barrel cortex in cPcdh-KO chimeric mice at P18–20. Scale bar: 100 μm. (**B**) A representative trace of action potentials evoked by depolarizing current injection (300 pA) in current clamp mode in cPcdh-KO neurons. (**C–E**) Box-and-whisker plot showing the value (median, 25th to 75th percentiles, minimum to maximum) of the resting membrane potential (RMP, C), the threshold to induce action potentials (firing threshold, D), and the input resistance (E) in cPcdh-KO neurons (yellow) and wild-type GFP-positive neurons (green, the same as shown in Fig. 2) at P18–20. No significant differences in these electrophysiological parameters between cPcdh-deficient cells and wild-type cells were observed; *P =* 0.45 (C), *P =* 0.21 (D), and *P =* 0.21 (E) (*t* test). The number of cells is indicated above each plot. (**F**) Percentage of synaptic connections between GFP-positive neuron pairs (P-P pairs) in P13–16 chimeric mice produced from different cPcdh-KO iPS cell lines (iPS-1 and iPS-2). No significant difference was observed between any of the two iPS cell lines (*P* =0.55, χ^2^ test). Numbers of recorded pairs are indicated on the bars. (PDF 295 kb)
Additional file 9:
**Figure S9.** The genotyping of the iPS cells by PCR experiments. Genotyping by PCR on DNA extracted from wild-type (WT), cPcdh-KO, and Dnmt3b-KO iPS cells. PCR with primer sets for detection of (**A**) cPcdh and (**B**) Dnmt3b genotype. M: DNA molecular weight marker. (PDF 547 kb)
Additional file 10:
**Table S1.** List of primer sequences. (PDF 114 kb)
Additional file 11:
**Table S2.** Alignment scores for the DNA sequence of each Pcdhγ gene versus the probes used for in situ hybridization. (PDF 27 kb)
Additional file 12:
**Table S3.** The combinations of mouse strains, staining methods, RNA probe label, and antibodies. (PDF 49 kb)
