# Supplementary figures and images for: Establishment of high reciprocal connectivity between clonal cortical neurons is regulated by the Dnmt3b DNA methyltransferase and clustered protocadherins
Source: BMC Biol. 2016 Dec 2;14:103. doi: 10.1186/s12915-016-0326-6 (PMC5133762; doi:10.1186/s12915-016-0326-6)

**A** P18-20 wild-type mice

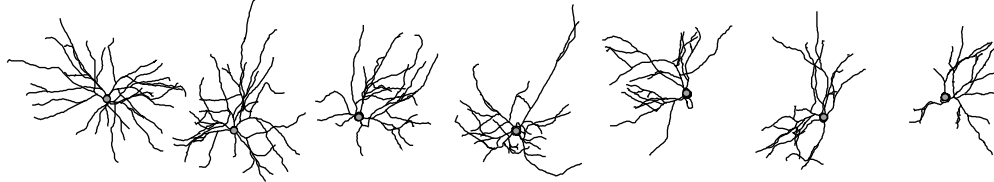

**B** P18-20 P-cell in wild-type chimeric mice

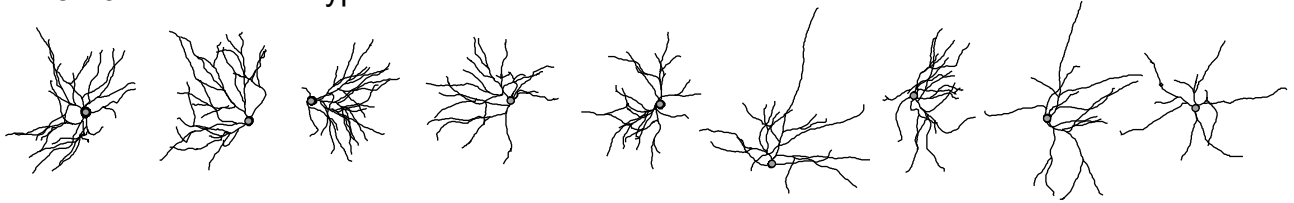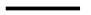

Additional file 1 Figure S1

Supplement: Additional file 1: — Figure S1. Dendritic morphology of recorded layer 4 neurons. (A, B) Traces from spiny stellate cells visualized by biocytin staining in layer 4 of the barrel cortex at P18–20. Images show seven neurons from wild-type nonchimeric mice (A), and nine GFP-positive neurons (P cells) from wild-type chimeric mice (B). Scale bar: 100 μm. (PDF 673 kb) [file 12915_2016_326_MOESM1_ESM.pdf]

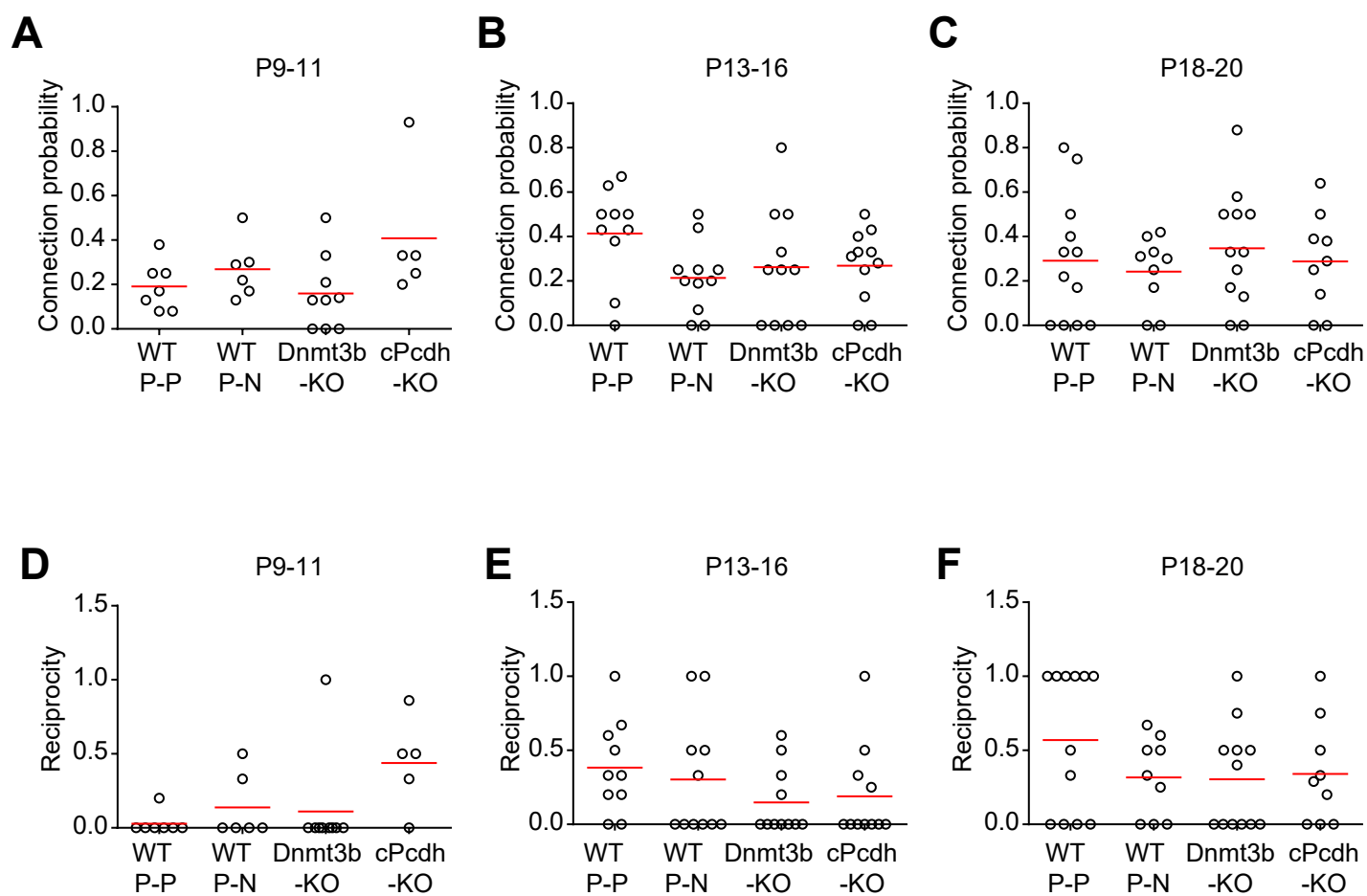

Additional file 2 Figure S2

Supplement: Additional file 2: — Figure S2. Synaptic connectivity obtained from each animal in four groups of neuron pairs. (A–C) Connection probability in wild-type (WT) P-P and P-N pairs, and Dnmt3b-KO and cPcdh-KO neuron pairs at P9–11 (A), P13–16 (B), and P18–20 (C). (D–F) Reciprocity in WT P-P and P-N pairs, and Dnmt3b-KO and cPcdh-KO neuron pairs at P9–11 (D), P13–16 (E), and P18–20 (F). Each symbol indicates an average value obtained from each animal. (PDF 416 kb) [file 12915_2016_326_MOESM2_ESM.pdf]

# **A** P13-16 stellate cells in wild-type chimeric mice

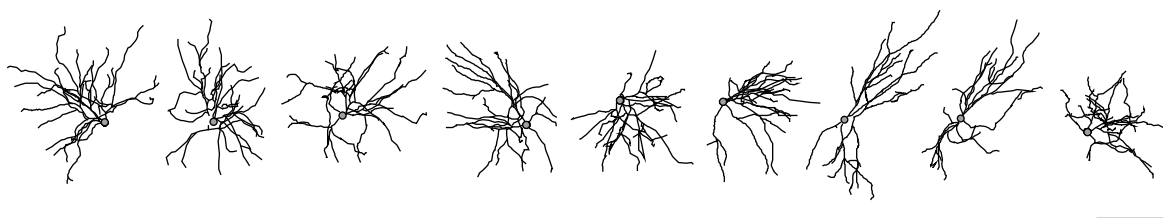

**B**

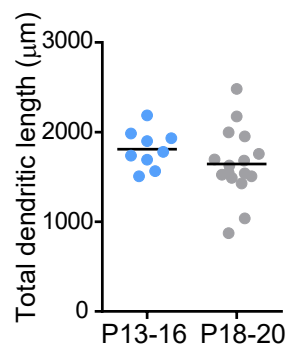

**C**

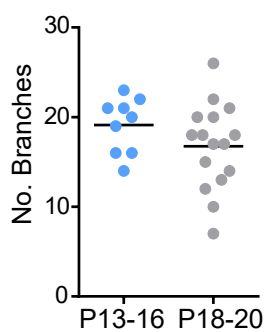

**D**

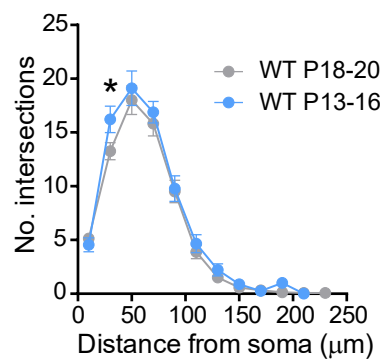

**E**

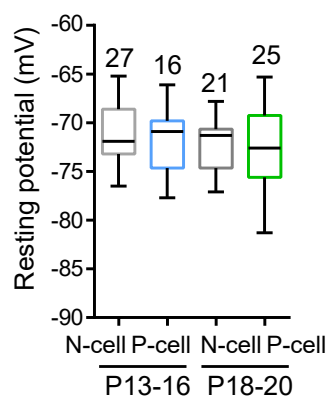

**F**

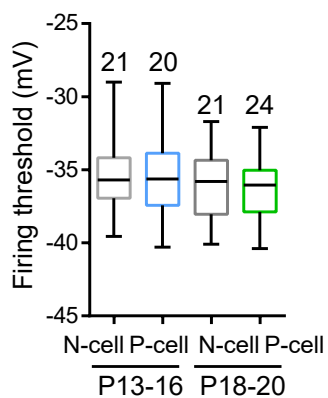

**G**

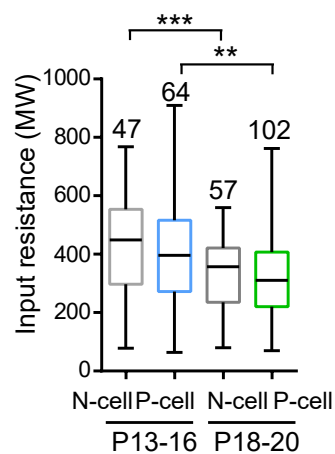

Additional file 3 Figure S3

Supplement: Additional file 3: — Figure S3. Comparison of morphological and electrophysiological properties of layer 4 neurons between P13–16 and P18–20 chimeric mice. (A) Traces of spiny stellate cells sampled from P13–16 wild-type chimeric mice (n = 9 cells, n = 8 barrels, n = 3 mice). Scale bar: 100 μm. (B–D) Comparison of the dendritic morphology between P13–16 (n = 9) and P18–20 (n = 16) chimeric mice. No significant differences in the total dendritic length (P = 0.26, t test, B) or the number of branches (P = 0.20, C) were observed, but there was a difference in the number of intersections near soma (*P = 0.046, D). A bar indicates the mean (B, C). Data presented as mean ± SEM in D. (E–G) Comparison of the GFP-negative neurons (N-cell) or GFP-positive neurons (P-cell) between P13–16 and P18–20 chimeric mice in the resting potential (P = 0.30 for GFP-negative neurons, P = 0.36 for GFP-positive neurons, t test, E), firing threshold (P = 0.44 for GFP-negative neurons, P = 0.20 for GFP-positive neurons, F), and input resistance (***P = 0.0007 for GFP-negative neurons, **P = 0.003 for GFP-positive neurons, G). Number of analyzed cells is shown above each box-and-whisker plot (median, 25th to 75th percentiles, minimum to maximum) (E–G). (PDF 636 kb) [file 12915_2016_326_MOESM3_ESM.pdf]

**A**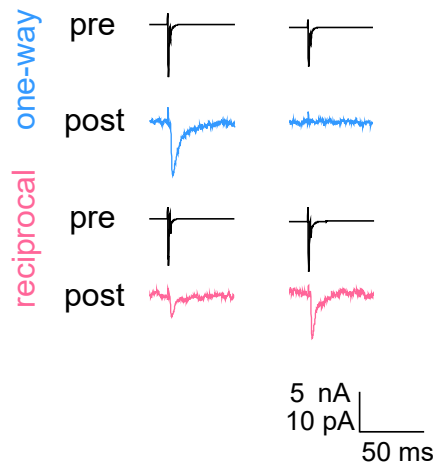**B**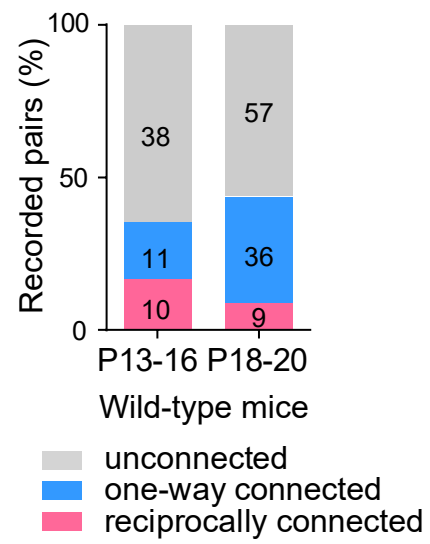

Additional file 4 Figure S4

Supplement: Additional file 4: — Figure S4. Synaptic connections in wild-type nonchimeric mice. (A) Representative average (n = 20) traces of presynaptic spikes (pre) and resultant excitatory postsynaptic currents (post) between layer 4 neuron pairs with a one-way (upper) or reciprocal (lower) connection in wild-type nonchimeric mice (C57BL/6 mice) at P18–20. (B) Percentage of neuron pairs with synaptic connections. There was no significant difference in the connectivity between the neuron pairs in wild-type nonchimeric mice and the nonclonal neuron pairs in wild-type chimeric mice at the same age (P > 0.17, χ2 test). Numbers of recorded pairs are indicated on the bars. (PDF 575 kb) [file 12915_2016_326_MOESM4_ESM.pdf]

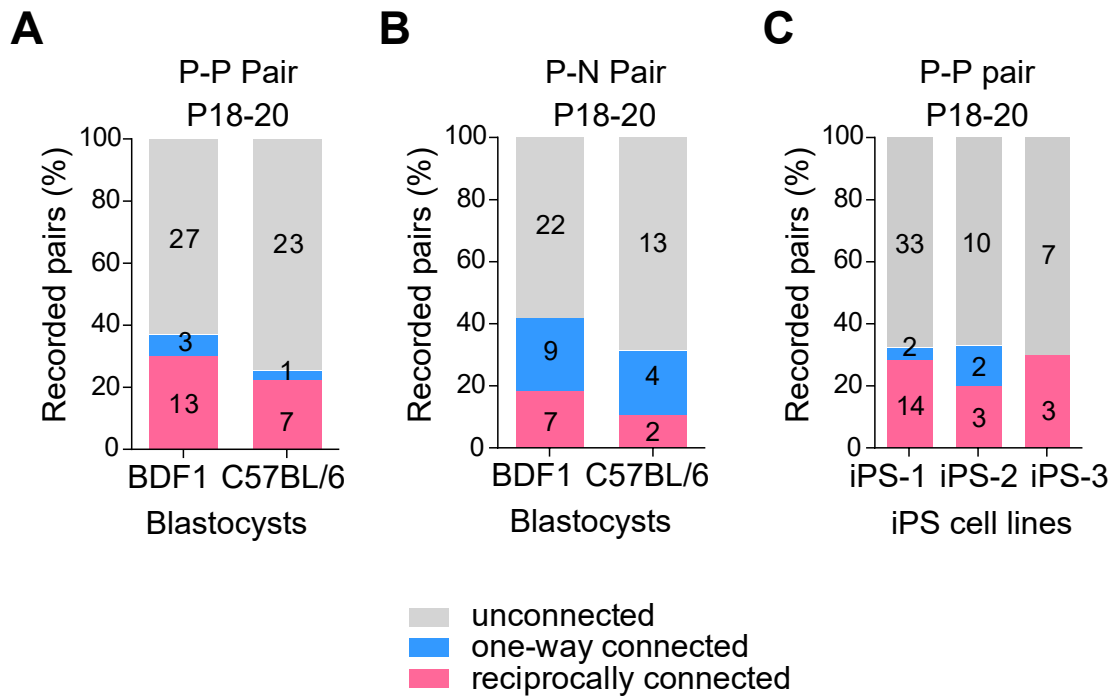

Additional file 5 Figure S5

Supplement: Additional file 5: — Figure S5. Synaptic connectivity is not affected by the mouse strain used for the blastocysts or iPS cell lines. (A) Percentage of synaptic connections between clonal neuron pairs (P-P pairs) in P18–20 wild-type chimeric mice produced with blastocysts prepared from BDF1 or C57BL/6 mice; iPS cells were produced using C57BL/6 mice. There was no significant difference in the connectivity between the two strains (P = 0.55, χ2 test). (B) Similar to A, but between nonclonal neuron pairs (P-N pairs). There was no significant difference in the connectivity between the two strains (P = 0.68, χ2 test). (C) Percentage of synaptic connections between GFP-positive neuron pairs (P-P pairs) in P18–20 chimeric mice produced from different wild-type iPS cell lines (iPS-1, iPS-2, and iPS-3). No significant difference was observed between any of the three iPS cell lines (P = 0.59, χ2 test). Numbers of recorded pairs are indicated on the bars. (PDF 540 kb) [file 12915_2016_326_MOESM5_ESM.pdf]

**A** Dnmt3b-KO cell

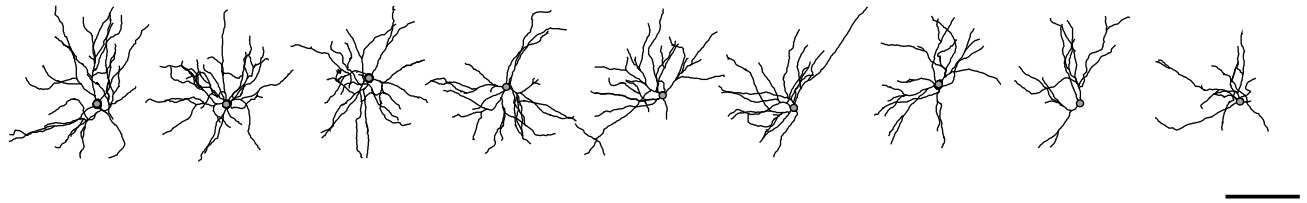

**B** Dnmt3b-KO cell

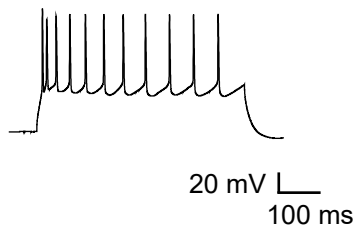

**C**

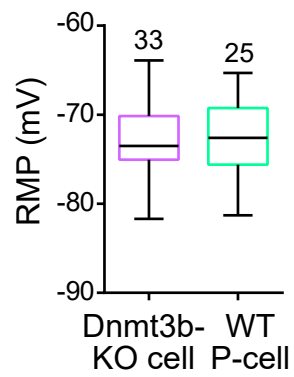

**D**

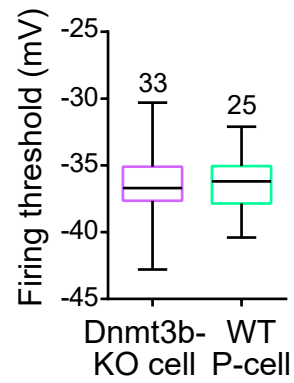

**E**

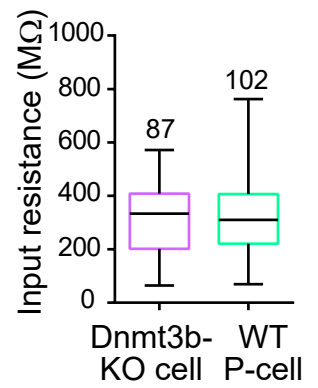

Additional file 6 Figure S6

Supplement: Additional file 6: — Figure S6. Morphological and electrophysiological properties of Dnmt3b-KO neurons. (A) Traces from iPS cell-derived Dnmt3b-KO spiny stellate cells in layer 4 of the barrel cortex in Dnmt3b-KO chimeric mice at P18–20. The neurons were stained by biocytin. Scale bar: 100 μm. (B) A representative trace of action potentials evoked by depolarizing current injection (300 pA) in current clamp mode in Dnmt3b-KO neurons. (C–E) Box-and-whisker plot showing the value (median, 25th to 75th percentiles, minimum to maximum) of the resting membrane potential (RMP, C), the threshold to induce action potentials (firing threshold, D), and the input resistance (E) in Dnmt3b KO neurons (purple) and wild-type GFP-positive neurons (green, the same as shown in Fig. 2) at P18–20. No significant differences in electrophysiological parameters between Dnmt3b-deficient cells and wild-type cells were observed; P = 0.72 (C), P = 0.71 (D), and P = 0.68 (E) (t test). The numbers of cells are indicated above the box-and-whisker plot. (PDF 728 kb) [file 12915_2016_326_MOESM6_ESM.pdf]

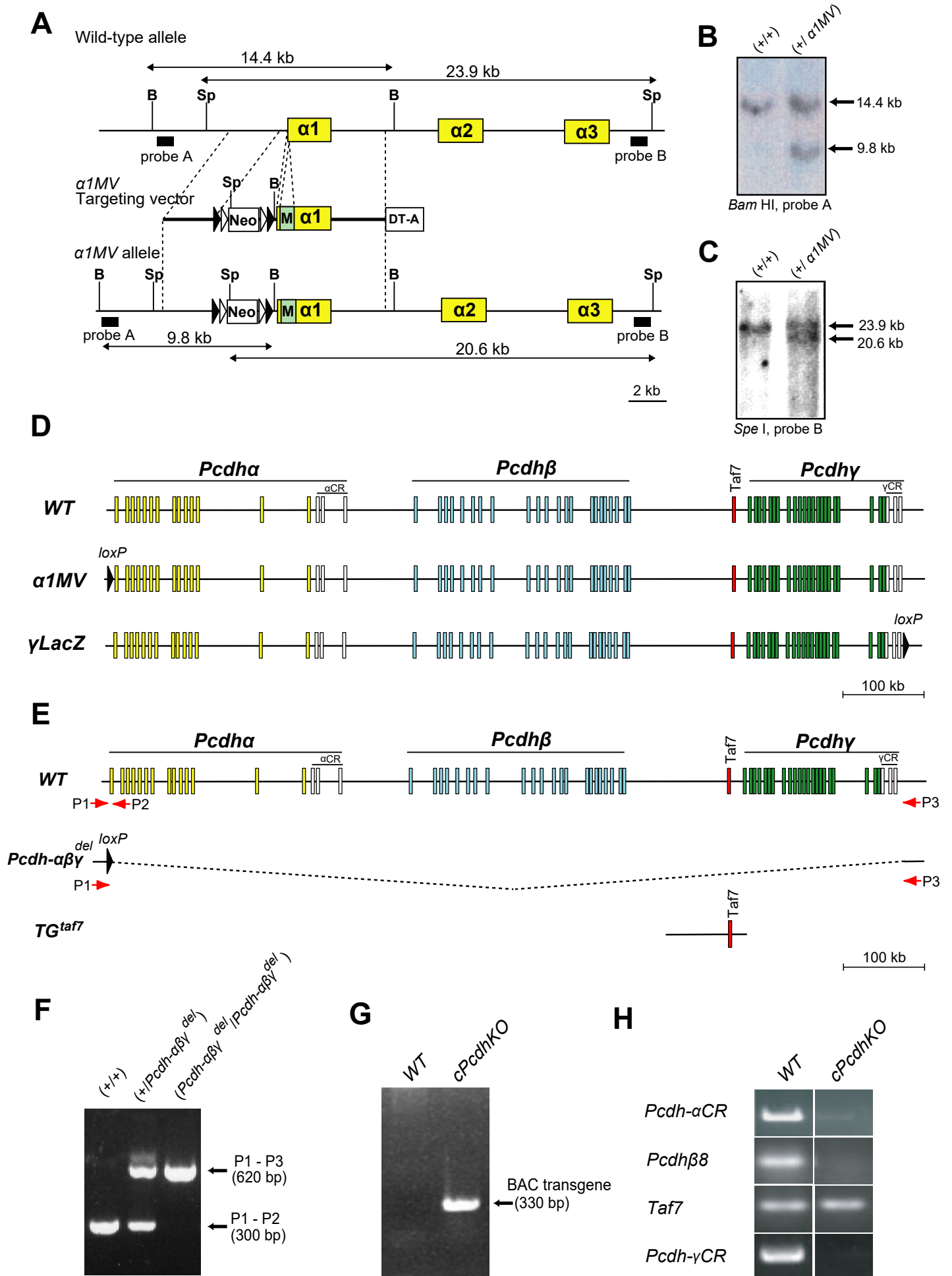

Additional file 7 Figure S7

Supplement: Additional file 7: — Figure S7. Generation of cPcdh KO mice that have a Pcdh-abg del/del allele and a TG taf7 BAC transgene. (A) Schematic diagram of the a1MV targeting constructs. Filled triangles: loxP sites; open triangles: frt sites; M: Myc-tagged venus fluorescent protein gene; Neo: neomycin-resistance gene; DT-A: diphtheria toxin A fragment gene; α1: cPchd-a1 exon; α2: cPchd-a2 exon; α3: cPchd-a3 exon; B: BamHI; Sp: SpeI. (B, C) Southern blotting of homologous recombinant ES cells digested by BamHI, with Probe A (B), and digested by SpeI, with Probe B (C). (D) Genetic structures of loxP site insertions. The loxP sites were inserted 5′ of the Pcdh-α cluster and 3′ of the Pcdh-γ cluster in a1MV and gLacZ mutant mice, respectively. (E) Genetic structures of Pcdhαβγ del mice and TG taf7 BAC transgenic mice. Arrows indicate the primer positions used for genotyping. αCR: cPcdh-α constant region; γCR: cPcdh-γ constant region. (F, G) Genotyping by PCR of cPcdh-KO mutants with the Pcdh-αβγ del allele and the TG taf7 BAC transgene. (H) Expression of Pcdh-α, Pcdh-β, Pcdh-γ, and taf7 genes in the E18.5 brain of wild-type and cPcdh-KO mice, by RT-PCR analysis. All of the genotyping and gene expression experiments yielded similar results. (PDF 869 kb) [file 12915_2016_326_MOESM7_ESM.pdf]

**A** cPcdh-KO cell

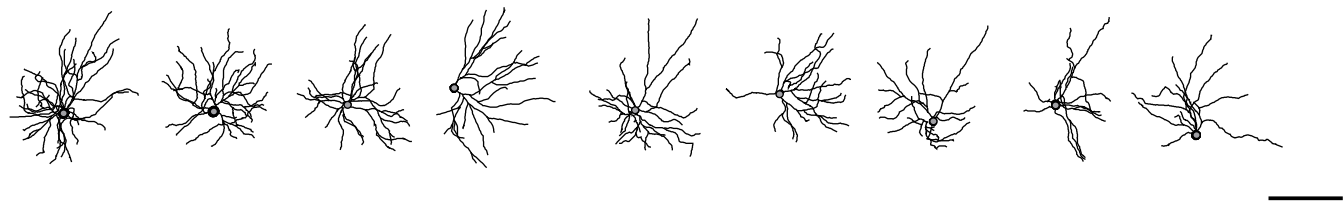

**B**

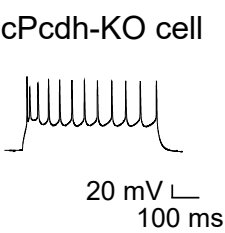

**C**

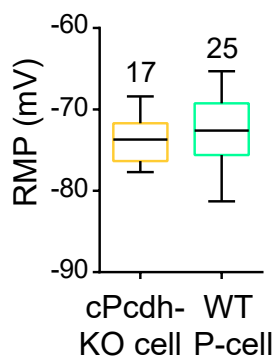

**D**

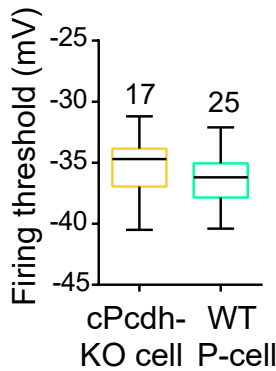

**E**

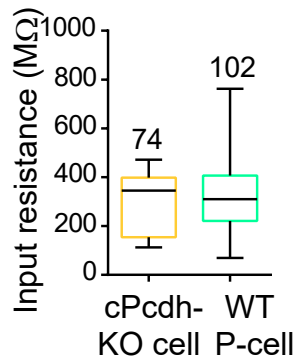

**F**

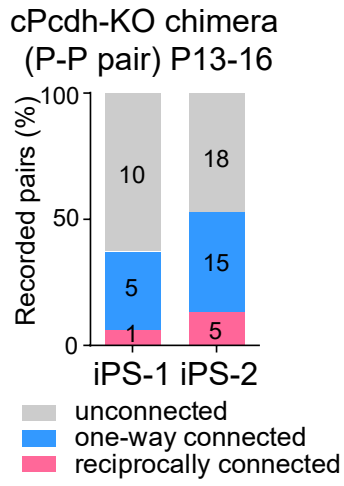

Additional file 8 Figure S8

Supplement: Additional file 8: — Figure S8. Morphological and electrophysiological properties of cPcdh-KO neurons. (A) Traces from iPS-derived tdTomato-positive spiny stellate cells in layer 4 of the barrel cortex in cPcdh-KO chimeric mice at P18–20. Scale bar: 100 μm. (B) A representative trace of action potentials evoked by depolarizing current injection (300 pA) in current clamp mode in cPcdh-KO neurons. (C–E) Box-and-whisker plot showing the value (median, 25th to 75th percentiles, minimum to maximum) of the resting membrane potential (RMP, C), the threshold to induce action potentials (firing threshold, D), and the input resistance (E) in cPcdh-KO neurons (yellow) and wild-type GFP-positive neurons (green, the same as shown in Fig. 2) at P18–20. No significant differences in these electrophysiological parameters between cPcdh-deficient cells and wild-type cells were observed; P = 0.45 (C), P = 0.21 (D), and P = 0.21 (E) (t test). The number of cells is indicated above each plot. (F) Percentage of synaptic connections between GFP-positive neuron pairs (P-P pairs) in P13–16 chimeric mice produced from different cPcdh-KO iPS cell lines (iPS-1 and iPS-2). No significant difference was observed between any of the two iPS cell lines (P =0.55, χ2 test). Numbers of recorded pairs are indicated on the bars. (PDF 295 kb) [file 12915_2016_326_MOESM8_ESM.pdf]

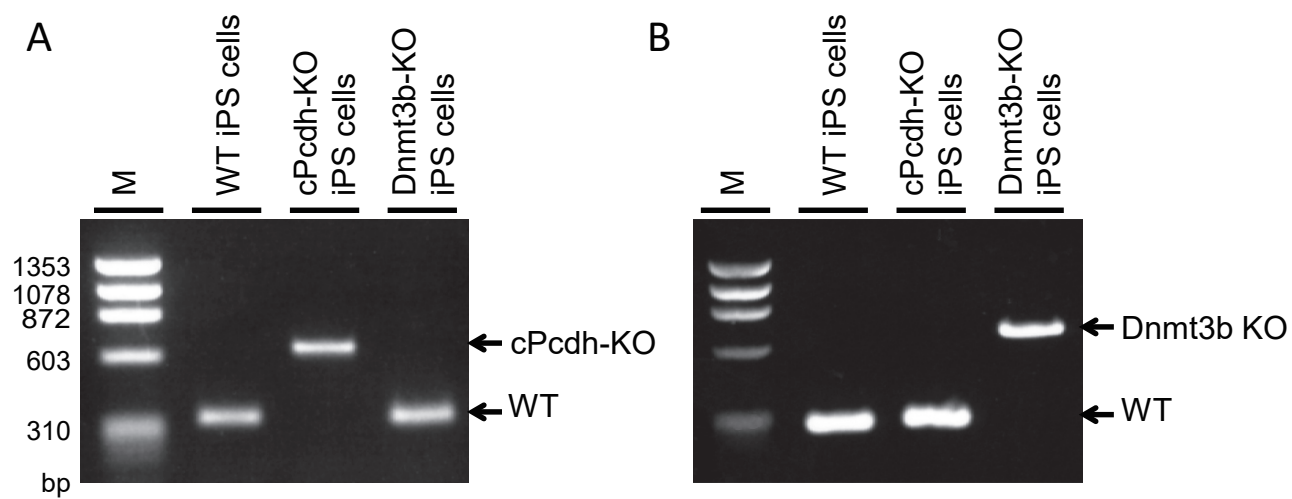

Additional file 9 Figure S9

Supplement: Additional file 9: — Figure S9. The genotyping of the iPS cells by PCR experiments. Genotyping by PCR on DNA extracted from wild-type (WT), cPcdh-KO, and Dnmt3b-KO iPS cells. PCR with primer sets for detection of (A) cPcdh and (B) Dnmt3b genotype. M: DNA molecular weight marker. (PDF 547 kb) [file 12915_2016_326_MOESM9_ESM.pdf]
