# Supplementary material for: Establishment of high reciprocal connectivity between clonal cortical neurons is regulated by the Dnmt3b DNA methyltransferase and clustered protocadherins
Source: BMC Biol. 2016 Dec 2;14:103. doi: 10.1186/s12915-016-0326-6 (PMC5133762; doi:10.1186/s12915-016-0326-6)
Supplement: Additional file 10: — Table S1. List of primer sequences. (PDF 114 kb) [file 12915_2016_326_MOESM10_ESM.pdf]

Additional file 10: Table S1.

List of primer sequences

<For genotyping of cPcdh KO mutant mice>

P1: 5’- GCTTATTTGCTAGAACGTCC - 3’
P2: 5' - CGGAGCCTGGAAAACAGCAT - 3'
P3: 5’- GCTTATTTGCTAGAACGTCC - 3’
P4: 5' - GCTCCTGATTGAATTTGCC - 3'
P5: 5’- CCGCTTCCTCGTGCTTTAC- 3’
P6: 5' - TTTGGTGCATCCATTTGGAGTGTGG - 3'
P7: 5’- TGATGTGGGTCTGGTTTCC - 3’
P8: 5' - CCAAGGCGACAGAGAAGGAAG - 3'

<For genotyping of cPcdh-KO iPS cells>

v1up 300-F GCTTATTTGCTAGAACGTCC
acKO wildR CGGAGCCTGGAAAACAGCAT
EN-R CCAAGGCGACAGAGAAGGAAG

<For genotyping of Dnmt3b-KO iPS cells>

OM159F AGAGCACTGCACCACTACTGCTGGA
OM147R CAGGTCAGACCTCTCTGGTGACAAG
OM146F GAACTTGGTCTGCAGGACGATCGCT

Construction of targeting vectors

α1MV targeting vector

a1MVA-F: 5' - CGGTCGACGTCATGTACAAGTTCTATGCC - 3'
a1MVA-R: 5' - CGGTCGACGATCATACTTTGCACCTTCATG - 3'
a1MVB-F: 5' - CGGGATCCTTGGTGTGACAGCGATACGG - 3'
a1MVB-R:
5' - CCATCGATCAGATCCTCTTCAGATGAGTTTCTGCTCAATCTGGCCGCTCCCTGCC - 3'
a1MVC-F: 5' - CCATCGATCGATCTCTCGAGATGGTGAGCAAGGGCGAGG - 3'
a1MVC-R: 5' - CCTGCAGGCTTGTACAGCTCGTCCATGC - 3'
a1MVD-F: 5' - CCATCGATCCTGCAGGCCAGATTCATTACTCTGTCCC - 3'
a1MVD-R: 5' - CGAGCTCGCGTCGACGTTCGTCTGTTGTCTCTACC - 3'
a1MVE-F: 5' - CCCAAGCTTAATGCGGCCGCCAACTAGCTCTGTAGACCAGG - 3'
a1MVE-R: 5' - ACGCGTCGACGCATTTATCATTCTGTCATGAGGG - 3'
a1MVF-F : 5' - ACGCGTCGACAGGTCGCTTGACTCAAGGTTTCC - 3'
a1MVF- R: 5' - CTAGCTAGCGACCTGGATTTCATAGGATGTCC - 3'

Isolation of probes for Southern hybridization analysis

probe A

F: 5' - GAAATGAAATTTGATAGATGG - 3'
R: 5' - AATACAACACATTTCCAACC - 3'

probe B

F: 5'- ACAGAGAGGACATTCCTG - 3'
