# Supplementary material for: Establishment of high reciprocal connectivity between clonal cortical neurons is regulated by the Dnmt3b DNA methyltransferase and clustered protocadherins
Source: BMC Biol. 2016 Dec 2;14:103. doi: 10.1186/s12915-016-0326-6 (PMC5133762; doi:10.1186/s12915-016-0326-6)
Supplement: Additional file 11: — Table S2. Alignment scores for the DNA sequence of each Pcdhγ gene versus the probes used for in situ hybridization. (PDF 27 kb) [file 12915_2016_326_MOESM11_ESM.pdf]

Additional file 11: Table S2.

Alignment scores for the DNA sequence of each Pcdhγ gene versus the probes used for in situ hybridization.

probe / gene	PcdhgA3	PcdhgA7
PcdhgA1	62	64
PcdhgA2	67	66
PcdhgA3	100	64
PcdhgA4	64	65
PcdhgA5	61	64
PcdhgA6	60	66
PcdhgA7	64	100
PcdhgA8	62	62
PcdhgA9	63	65
PcdhgA10	64	68
PcdhgA11	60	65
PcdhgA12	62	66
PcdhgB1	54	56
PcdhgB2	55	53
PcdhgB4	49	52
PcdhgB5	54	53
PcdhgB6	56	54
PcdhgB7	55	58
PcdhgB8	55	57
PcdhgC3	54	57
PcdhgC4	53	55
PcdhgC5	54	57
