# Supplementary material for: Establishment of high reciprocal connectivity between clonal cortical neurons is regulated by the Dnmt3b DNA methyltransferase and clustered protocadherins
Source: BMC Biol. 2016 Dec 2;14:103. doi: 10.1186/s12915-016-0326-6 (PMC5133762; doi:10.1186/s12915-016-0326-6)
Supplement: Additional file 12: — Table S3. The combinations of mouse strains, staining methods, RNA probe label, and antibodies. (PDF 49 kb) [file 12915_2016_326_MOESM12_ESM.pdf]

Additional file 12: Table S3. The combinations of mouse strains, staining methods, RNA probe label, and antibodies.

**Mouse**		**cPcdh-KO chimera**	**Dnmt3b-KO chimera**	**Wt GFP-chimera**
**Staining method**		dual ISH	ISH/IF	ISH/IF
**Probe**	**Fluorescent**	tdTomato	cPcdhs*	cPcdhs*
	**DIG**	cPcdhs*	n/a	n/a
**Primary antibody**		n/a	Chicken anti-GFP Ab	Chicken anti-GFP Ab
**Secondary antibody**		n/a	Goat anti-Chicken IgG Ab Alexa488	Goat anti-Chicken IgG Ab Alexa488

*cPcdhs; *PcdhγA3* and *PcdhγA7*
